# Evidence against the “normalization” prediction of the early brain overgrowth hypothesis of autism

**DOI:** 10.1186/s13229-020-00353-2

**Published:** 2020-06-18

**Authors:** Lisa D. Yankowitz, John D. Herrington, Benjamin E. Yerys, Joseph A. Pereira, Juhi Pandey, Robert T. Schultz

**Affiliations:** 1 Center for Autism Research, Children’s Hospital of Philadelphia, 2716 South St, Philadelphia, PA 19104 USA; 2 Department of Psychology, University of Pennsylvania, 425 S. University Ave, Philadelphia, PA 19104 USA; 3 Department of Psychiatry, Perelman School of Medicine, University of Pennsylvania, 3400 Civic Center Blvd, Philadelphia, PA 19105 USA; 4 Harvard Medical School, 25 Shattuck St, Boston, MA 02115 USA; 5 Department of Pediatrics Perelman School of Medicine, University of Pennsylvania, 3400 Civic Center Blvd, Philadelphia, PA 19105 USA

**Keywords:** Autism, Brain volume, Structural imaging, MRI, IQ, Adolescent

## Abstract

**Background:**

The frequently cited Early Overgrowth Hypothesis of autism spectrum disorder (ASD) postulates that there is overgrowth of the brain in the first 2 years of life, which is followed by a period of arrested growth leading to normalized brain volume in late childhood and beyond. While there is consistent evidence for early brain overgrowth, there is mixed evidence for normalization of brain volume by middle childhood. The outcome of this debate is important to understanding the etiology and neurodevelopmental trajectories of ASD.

**Methods:**

Brain volume was examined in two very large single-site samples of children, adolescents, and adults. The primary sample comprised 456 6–25-year-olds (ASD *n* = 240, typically developing controls (TDC) *n* = 216), including a large number of females (*n* = 102) and spanning a wide IQ range (47–158). The replication sample included 175 males. High-resolution T1-weighted anatomical MRI images were examined for group differences in total brain, cerebellar, ventricular, gray, and white matter volumes.

**Results:**

The ASD group had significantly larger total brain, cerebellar, gray matter, white matter, and lateral ventricular volumes in both samples, indicating that brain volume remains enlarged through young adulthood, rather than normalizing. There were no significant age or sex interactions with diagnosis in these measures. However, a significant diagnosis-by-IQ interaction was detected in the larger sample, such that increased brain volume was related to higher IQ in the TDCs, but not in the ASD group. Regions-of-significance analysis indicated that total brain volume was larger in ASD than TDC for individuals with IQ less than 115, providing a potential explanation for prior inconsistent brain size results. No relationships were found between brain volume and measures of autism symptom severity within the ASD group.

**Limitations:**

Our cross-sectional sample may not reflect individual changes over time in brain volume and cannot quantify potential changes in volume prior to age 6.

**Conclusions:**

These findings challenge the “normalization” prediction of the brain overgrowth hypothesis by demonstrating that brain enlargement persists across childhood into early adulthood. The findings raise questions about the clinical implications of brain enlargement, since we find that it neither confers cognitive benefits nor predicts increased symptom severity in ASD.

## Introduction

Autism spectrum disorder (ASD) is a neurobiologically-based, highly heritable condition [4, 81], but the brain bases of ASD have proven complex and difficult to characterize. An early neurobiological insight was Leo Kanner’s observation that the majority of his autistic patients^1^ had enlarged head sizes [43]. Numerous studies have since confirmed greater head circumference in ASD compared to typically developing control (TDC) samples, especially in studies with large sample sizes (e.g., [17]; for review see [68]). Piven and colleagues were the first to report increased brain volume in ASD compared to controls using MRI [61], which has been replicated in several studies [32, 33, 57]. However, not all imaging studies have found significant enlargement [3, 35, 37, 52, 70, 80].

One highly cited hypothesis—the Early Brain Overgrowth hypothesis [20]—accounts for inconsistencies in volumetric studies of ASD by postulating (1) average brain size at birth, followed by (2) periods of accelerated growth over the next 2 years, and then (3) deceleration of brain growth, equalizing volumes between groups by middle to late childhood. Support for the first two predictions (average size at birth followed by overgrowth) has been strong and consistent. Head circumference, which correlates highly with brain volume in newborns [49], has been observed to be normal at birth in most individuals who go on to develop ASD [21]. Moreover, brain volume appears to remain typical through 6 months of age in infants later diagnosed with ASD [38]. A recent longitudinal MRI study found a significantly greater rate of growth of total brain volume (TBV) between 12 and 24 months in ASD, resulting in significantly greater TBV in the ASD group at age 2 [39]. Consistent reports of larger TBV in young children with ASD [15, 22, 40, 75] also support the second prediction of the early overgrowth hypothesis: accelerated growth in the first 2 years.

The third prediction—arrested growth leading to normalization—has proven controversial. Several studies have found no difference in total brain volume between ASD and TDC groups in school-age [3, 37, 52, 80] and/or adulthood [35, 70], and a 2005 meta-analysis only supported overgrowth in 2-to-5-year-old autistic children, but not in children older than 6 years [63]. Nevertheless, many studies of school-age to adult samples have identified larger brain volumes in the ASD group [32, 33, 57, 61]. Two more-recent meta-analyses (2008 and 2015) support continued enlargement in school-age through adulthood [68, 76]. In a study of 1881 families of autistic 4–18-year-olds, affected probands had larger head circumference than their unaffected siblings, with an effect of 0.2 cm [17]. Although not a direct measure of brain volume, this study suggests that when appropriately controlling for sex, age, height, weight, and genetic ancestry, head size (an adequate predictor of brain volume, [6]), remains enlarged in ASD.

If overgrowth does persist in ASD, it is not clear which tissue types drive these differences. Some research suggests an imbalance of gray matter (GM) and white matter (WM). However, both increased GM relative to WM [12] and increased WM relative to GM [41] have been noted. Differences in ventricle size have also been noted, including enlarged third ventricles [36], and that ventricular enlargement in neonatal low-birth weight babies relates to a seven-fold increased risk of ASD development [54].

Previous work investigating brain volume in school age and beyond is limited by small sample sizes. For example, in the most recent meta-analysis [68], ASD samples ranged from 6 to 121 individuals, with a median of 20. Given the well-known heterogeneity in ASD of core symptom severity, intellectual abilities, and co-occurring psychiatric conditions [5, 51, 72], small samples have decreased statistical power to detect true effects and increased chance of studying biased groups, producing results that are harder to replicate [14]. Meta-analytic efforts mitigate some concerns related to sample size, but sampling error in small original studies can result in biased meta-analytic estimates [48], and publication bias and selective reporting lead to biased effect size estimates in meta-analysis that are difficult to correct [47]. While multi-site network studies producing publicly available datasets, such as the Autism Brain Imaging Data Exchange (ABIDE [24];), are pushing the field toward ever-larger datasets, inter-site and inter-scanner differences contribute significant noise to these data, which may be nonlinear [31]. This between-scanner noise, when random, limits the ability to find group differences [2, 78], and when systematic, biases observed effects.

Small sample sizes also limit the investigation of important individual differences, such as IQ, sex, and ASD symptom severity. Both sex and IQ are known correlates of brain volume (with larger brains in males and individuals with higher IQ [64, 91], and have known clinical relationships with ASD. Approximately half of autistic children have IQ more than one standard deviation below the mean [5]. ASD is four times more prevalent in boys than girls, who are often disproportionately under-represented or excluded from imaging studies. Some studies of brain structure and white matter tracts [8, 10], and functional connectivity [92] have suggested interactions between sex and diagnosis, but more systematic study of sex differences is needed. Moreover, efforts to associate brain volume with core ASD symptom severity have produced mixed results [1, 68].

To assess the prediction of the Early Brain Overgrowth hypothesis that brain volume normalizes by school age and adolescence in ASD, we investigated the relationships of diagnosis, age, sex, IQ, and core ASD symptom severity with global brain volumes (i.e., total brain, gray matter, white matter, and ventricular volumes) in a large, diverse sample of children, adolescents, and adults. There are several important strengths to the samples examined in the present study. The samples are among the largest of their kind, including 456 individuals in the primary sample, and 175 in the replication. Crucially, within each sample, all individuals were characterized and imaged at the same site, using the same MRI scanner and scan sequence, eliminating sources of error variance that are present in large samples produced by combining data across research sites. The primary sample is particularly strong in terms of diversity of several key characteristics with potential etiological correlates in ASD, including a large number of females with ASD (43, which to our knowledge represents the largest single-site female structural MRI sample to date), an inclusive IQ range (47-158), and a wide age (6 to 25).

## Materials and methods

### Participants

Participants in the primary sample were selected from the larger group of individuals who had participated in any imaging study at CHOP’s Center for Autism Research between 2009 and 2015, from whom a structural anatomical image was acquired. For individuals with ASD, final diagnosis was made by expert clinical judgment using DSM-IV criteria using results from the Autism Diagnostic Observation Schedule [50] and the Autism Diagnostic Interview-Revised [67]. In keeping with DSM-5, all diagnostic subcategories (autism, Asperger’s, PDD-NOS) were pooled into a single ASD group in this study. Four hundred ninety-eight participants had structural scans. Nineteen of these were excluded due to bad scan quality, and 16 were excluded because they received a final diagnosis other than ASD. Seven more individuals were excluded for not having an IQ estimate, leaving a final sample of 456 individuals (see Table 1 and S1, Additional file 1 for demographic data, Figure S1, Additional file 1 for age distributions). Diagnostic groups did not differ significantly on mean age or height (in the subset of 281 individuals for whom height was available at the time of the MRI). Groups differed significantly on proportion of males, reflecting general population differences between ASD and TDC. Racial proportions differed significantly between groups, so sensitivity analyses entailed repeating all analyses within only the White participants.

**Table 1:** Demographic and clinical information for the primary and replication samples

	ASD	TDC	*t*-/*χ*^2^ value	*p* value
*N*	240	216		
Males (%)	197 (82.1)	157 (72.7)	5.8	0.016
Age years (SD) Range	13.0 (3.5) 6.4-25.9	13.1 (4.1) 6.3-25.6	− 0.43	0.67
IQ (SD) Range	100.9 (20.6) 47–158	113.0 (16.0) 67–155	− 7.0	< 0.001
SRS-II (SD) Range *N*	73.3 (9.0) 47–89 210			
ADOS CSS (SD) Range *N*	6.9 (2.2) 1–10 238			
SCQ (SD) Range N	20.9 (6.1) 0–36 233			
Yale				
*N* (all male)	86	89		
Age years (SD) Range	16.6 (9.1) 5.5–46.0	20.6 (9.2) 8.3–55.4	− 2.9	0.004
IQ (SD) Range	97.1 (23.1) 56–144	112.1 (19.6) 59–149	− 4.6	< 0.001

### Cognitive ability

Participants’ cognitive ability (“IQ”) was assessed with one of four standard instruments: the General Cognitive Ability score of the Differential Abilities Scale, Second Edition [26], or the Full Scale IQ of the Wechsler Intelligence Scale for Children, Fourth Edition [90], and the Wechsler Abbreviated Scale of Intelligence, First or Second Edition [88, 89]. The distribution of IQ is shown in Figure S1, Additional file 1. IQ in the ASD group (*M* = 100.9, SD = 20.6) was significantly lower than controls (*M* = 113.0, SD = 16.0, *t* = − 7.0, *p* < 0.001).

### Clinical severity

Clinical severity was assessed with three measures: The Social Responsiveness Scale–2 (SRS-2), a parent questionnaire assessing current ASD traits [19]; the Autism Diagnostic Observation Schedule Calibrated Severity Score (ADOS CSS), an estimate of severity based on clinician ratings [34]; and the Social Communication Questionnaire (SCQ), a parent questionnaire assessing lifetime symptom severity [66].

### Parental education

Socio-economic status (parental educational attainment, occupation, and income) is related to children’s neurocognitive functioning, mediated by brain structure, with increased educational attainment of parents predicting increased surface area and volume in children [55]. Because of the well-known relationships between parental education, brain structure, and cognitive functioning, we (1) tested whether these relationships were observed in our TDC sample, and (2) conducted exploratory analyses to examine these relationships in ASD. See Supplementary Methods, Additional file 1 for treatment of this variable.

### Replication sample participants and characterization

The replication sample consisted of an all-male cohort collected at Yale University. From 215 available participants, 40 were excluded due to poor scan quality, leaving a final sample of 175. Distributions of age and IQ within the groups are shown in Figure S2, Additional file 1 and Table 1. Yale sample participants were evaluated with the Wechsler Intelligence Scale for Children, Third Edition [86], Wechsler Abbreviated Scale of Intelligence, First Edition [88], or the Wechsler Adult Intelligence Scale, Revised or Third Edition [85, 87]. Diagnostic groups differed significantly on age (*t* = − 2.92, *p* < 0.05) and IQ (*t* = − 4.60, *p* < 0.001).

### Image acquisition

CHOP anatomical images were acquired on a Siemens 3T wide-bore Magnetom Verio Tim scanner with a 32-channel head coil and a Siemens MPRAGE sequence (0.9 × 0.41 × 0.41 mm, TR = 1900, TE = 2.54, flip angle = 9). Replication sample images were collected at Yale University on a GE Signa 1.5 T using a high resolution SPGR sequence (2 NEX, 1.2 mm^3^; TR = 24, TE = 5, flip angle = 45, matrix=192 × 256, FOV = 30 cm, 124 contiguous 1.2 mm thick sagittal images).

### Image processing

**Fig. 1:**
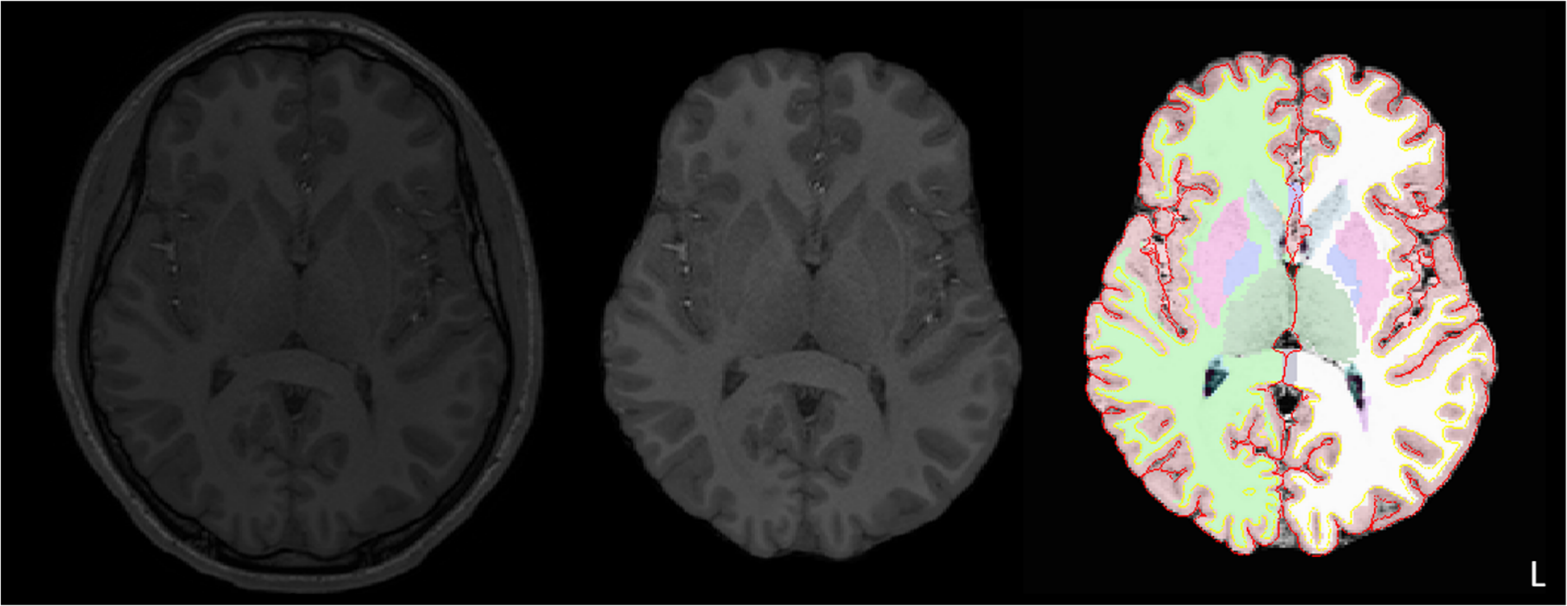
Raw T1-weighted images (left) were N3 bias corrected and skull-stripped, with manual corrections to ensure cortex was not removed (middle). Skull-stripped images were processed with Freesurfer with manual corrections (right), producing volume estimates

CHOP images were N3 bias corrected with ANTS [83] and brain extracted with LABEL ([71], see Fig. 1). Brain extractions were visually inspected, and manually edited with ITK-SNAP [93] if cortex was removed by the automated extraction. Yale images were intensity normalized using a histogram normalization procedure using the BioImage Suite Package [59]. Brain extraction was performed using BET (Brain Extraction Tool, S. M [74].), and conservatively thresholded to remove non-brain pixels only. Manual editing was performed to remove remaining non-brain tissue. Raters demonstrated excellent inter-rater reliability for brain volume (ICC = .99, *n* = 25).

For both datasets, segmentation of the volumes was performed by the Freesurfer image analysis suite (http://surfer.nmr.mgh.harvard.edu/, [23, 27–29]), producing total brain volume (TBV), gray matter volume (GMV), white matter volume (WMV), and ventricular volumes. To mitigate concerns that preprocessing techniques instantiated by different statistical packages show differential biases in comparing ASD and TDC volume [44], segmentations were visually inspected slice-by-slice (blind to all subject characteristics). Segmentation errors were manually edited using Freeview (e.g., dura labeled as gray matter, inaccurate identification of the gray/white or pial surface). Final segmentations were visually inspected and excluded if motion artifacts impacted segmentation quality, if a superior image was available for the participant (in the primary dataset), or if correspondence between Freesurfer’s total brain volume estimate and the total brain volume from gold standard manual tracing was exceptionally poor (in the replication dataset). See Supplementary Methods and Figure S3, Additional file 1 for reliability information and details about volume definitions.

For each volume measure, a regression model was tested including IQ, age, sex (CHOP only), diagnosis, the interaction of IQ and diagnosis, the interaction of age and diagnosis, and the interaction of sex and diagnosis (CHOP only). Nonsignificant interaction terms were removed to simplify the models and provide more precise estimates of the effect sizes of the main effects, with full models presented in Additional file 1 to illustrate null interaction findings. In the subset of individuals from the CHOP site for whom accurate height data was available, effects of height were also investigated. Effect sizes are reported as partial eta squared (partial *η*^2^) derived from equivalent ANOVAs. Partial *η*^2^ measures the proportion of variance in a dependent variable associated with each independent variable, with the effects of other independent variables and interactions partialled out, with suggested interpretive benchmarks of .01, .06, and .14 for small, medium, and large effects [65]. Estimates and standardized estimates from regressions are also included in the tables.

### Power

In CHOP models including all terms (4 main effects and 3 interactions), there is 80% power to detect effects of *f*^2^ = 0.03, where Cohen’s guidelines suggest that effects of *f*^2^ > 0.02 are small and *f*^2^ > 0.15 are medium. In Yale models with all terms (3 main effects and 2 interactions), there is 80% power to detect effects of *f*^2^ = 0.08. Thus, the CHOP sample is powered to detect small effects, and the Yale sample is powered to detect small-to-medium effects.

### Extreme size subgroup analysis

Some prior work has suggested that there is a higher rate of macrocephaly (head circumference above the 98th percentile) among autistic people. A recent meta-analysis found 15.7% of autistic participants had macrocephaly, and 9.1% showed brain overgrowth [68]. Higher rates of microcephaly have also been reported in ASD [30]. We conducted a post hoc analysis to explore the possibility that group-average differences in brain volume were driven by a subgroup of macrocephalic individuals in the ASD group. Within the TDC group, the mean and standard deviation of TBV were calculated within 3-year age bins separately by sex, and the number of individuals within the ASD group whose TBV exceeded 2 SD from the mean of their respective age/sex bin was examined. Individuals were excluded from this analysis when there were fewer than 2 TDC individuals within an age bin, because standard deviation could not be calculated. No individuals were excluded from the CHOP sample for this reason; 7 age bins including a total of 13 individuals were excluded from the Yale sample due to insufficient TDCs in the age bin.

## Results

### Group volume differences

**Fig. 2:**
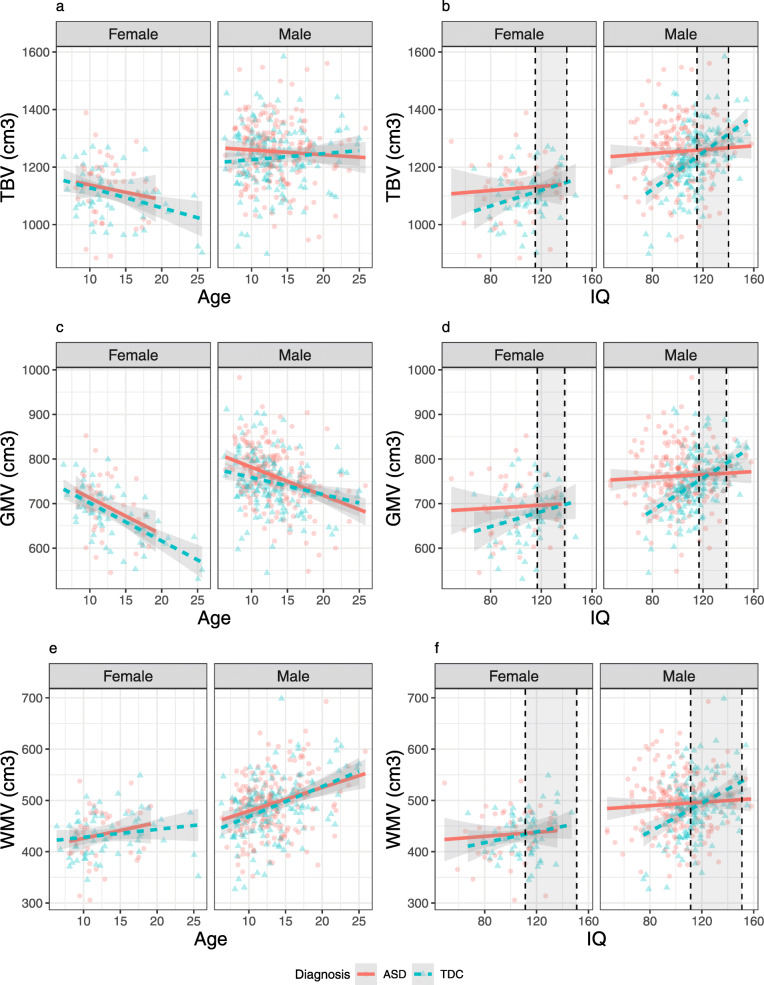
Relationships of IQ and age with total brain volume (**a**, **b**), gray matter volume (**c**, **d**), and white matter volume (**e**, **f**) in the primary sample, by diagnosis and sex. IQ shows a significant interaction with diagnosis predicting all three outcome measures (**b**, **d**, **e**). Age did not significantly predict TBV (**a**), negatively predicted GMV (**c**), and positively predicted WMV (**e**). Significant main effects of diagnosis and sex were observed in all 3 measures. Dashed lines indicate regions-of-significance, where the effect of diagnosis is not significant within the shaded region

Final models are presented in Table 2, with group means, standard deviations, and Cohen’s *d* effect size estimates presented in Table 3, and models including all non-significant interactions in Table S2, Additional file 1. Figure 2 graphically displays the relationships between TBV, GMV, WMV, and age and IQ, separated by diagnosis and sex in the CHOP sample. Diagnosis significantly predicted all brain tissue variables used in the analyses (TBV, GMV, WMV, cortical GMV, cortical WMV, cerebellar GMV, cerebellar WMV), except the ratio of GM to WM. All significant effects of diagnosis were in the direction of ASD showing larger volume than TDC. There also was a significant diagnosis-by-IQ interaction predicting TBV, GMV, WMV, cortical GMV, cortical WMV, and cerebellar GMV. There was no significant diagnosis-by-IQ interaction for cerebellar WMV.

**Table 2:** Models for primary sample. Uncorrected p-values are reported. One outlier was removed from the lateral ventricle model.

CHOP Models												
*Predictors*	*Estimates*	*std. Beta*	*p*	*partial η2*	*Estimates*	*std. Beta*	*p*	*partial η2*	*Estimates*	*std. Beta*	*p*	*partial η2*
	**TBV**				**GMV**				**WMV**			
Intercept	1034.67	-0.35	**<0.001**	0.69	698.45	-0.34	**<0.001**	0.77	337.69	-0.29	**<0.001**	0.48
IQ	1.52	0.25	**<0.001**	0.07	0.90	0.25	**<0.001**	0.08	0.62	0.20	**<0.001**	0.04
Age	-1.80	-0.06	0.143	0.00	-6.13	-0.33	**<0.001**	0.15	4.29	0.27	**<0.001**	0.09
Sex	85.43	0.73	**<0.001**	0.20	48.28	0.69	**<0.001**	0.21	37.13	0.62	**<0.001**	0.15
Diagnosis	-206.92	-0.25	**<0.001**	0.05	-130.81	-0.27	**<0.001**	0.07	-75.89	-0.17	**<0.001**	0.03
IQ*Diagnosis	1.67	0.28	**<0.001**	0.04	1.05	0.29	**<0.001**	0.05	0.62	0.20	**0.001**	0.02
R^2^ / R^2^ adjusted	0.290 / 0.282				0.377 / 0.370				0.280 / 0.272			
	**Cortical GMV**				**Cortical WMV**							
Intercept	532.98	-0.33	**<0.001**	0.72	314.27	-0.30	**<0.001**	0.47				
IQ	0.75	0.25	**<0.001**	0.07	0.61	0.21	**<0.001**	0.05				
Age	-5.66	-0.36	**<0.001**	0.17	3.80	0.26	**<0.001**	0.08				
Sex	38.27	0.64	**<0.001**	0.18	35.57	0.63	**<0.001**	0.16				
Diagnosis	-112.55	-0.26	**<0.001**	0.07	-72.09	-0.17	**<0.001**	0.03				
IQ*Diagnosis	0.91	0.30	**<0.001**	0.06	0.59	0.20	**0.001**	0.02				
R^2^ / R^2^ adjusted	0.370 / 0.363				0.276 / 0.268							
	**Cerebellum**				**Cerebellar GMV**				**Cerebellar WMV**			
Intercept	128.62	-0.27	**<0.001**	0.66	105.20	-0.27	**<0.001**	0.68	23.42	-0.15	**<0.001**	0.35
IQ	0.13	0.17	**<0.001**	0.03	0.11	0.19	**<0.001**	0.04	0.02	0.06	0.178	0.00
Age	0.01	0.00	0.929	0.00	-0.48	-0.16	**<0.001**	0.03	0.49	0.37	**<0.001**	0.14
Sex	8.50	0.58	**<0.001**	0.12	6.94	0.59	**<0.001**	0.13	1.57	0.31	**<0.001**	0.04
Diagnosis	-15.91	-0.23	**0.004**	0.02	-12.11	-0.26	**0.005**	0.02	-3.80	-0.08	**0.046**	0.01
IQ*Diagnosis	0.12	0.16	**0.020**	0.01	0.09	0.14	**0.029**	0.01	0.03	0.12	0.065	0.01
R^2^ / R^2^ adjusted	0.176 / 0.167				0.213 / 0.204				0.189 / 0.180			
	**Lateral Ventricles**				**Third Ventricles**				**GMV:WMV Ratio**			
Intercept	7.28	-0.04	**<0.001**	0.03	0.75	-0.05	**<0.001**	0.14	1.93	0.05	**<0.001**	0.87
IQ	-0.01	-0.02	0.669	0.00	-0.00	-0.00	0.993	0.00	-0.00	-0.02	0.564	0.00
Age	0.28	0.19	**<0.001**	0.03	0.00	0.01	0.827	0.00	-0.03	-0.67	**<0.001**	0.46
Sex	0.49	0.08	0.288	0.00	0.03	0.12	0.119	0.01	-0.02	-0.13	**0.033**	0.01
Diagnosis	-0.83	-0.14	**0.042**	0.01	0.09	-0.20	0.148	0.00	-0.01	-0.05	0.334	0.00
Age*Diagnosis					-0.01	-0.16	**0.017**	0.01				
R^2^ / R^2^ adjusted	0.049 / 0.040				0.040 / 0.029				0.464 / 0.459

**Table 3:** Mean and standard deviation of volumes in each group in the CHOP sample, and Cohen’s *d* for the difference between groups. Note that groups are not matched for age, sex, and IQ, and that the Cohen’s *d* effect size estimate does not account for these factors

CHOP group means			
	ASD mean (SD)	TDC mean (SD)	Cohen’s *d*
TBV	1231.06 (117.58)	1197.48 (115.7)	0.29
GMV	749.45 (68.59)	726.56 (69.86)	0.33
WMV	482.64 (60.58)	471.94 (58.46)	0.18
Cortical GMV	567.24 (58.52)	549.15 (59.31)	0.31
Cortical WMV	450.07 (57.18)	439.86 (55.38)	0.18
Cerebellum	148.65 (15.14)	144.29 (13.86)	0.3
Cerebellar GMV	116.07 (11.96)	112.22 (10.99)	0.34
Cerebellar WMV	32.57 (5.07)	32.07 (5.03)	0.1
Lateral ventricles	11.39 (7.03)	9.88 (5.76)	0.23
Third ventricle	0.81 (0.28)	0.73 (0.27)	0.3

To further understand this interaction, the regions of significance of the diagnosis-by-IQ interaction were evaluated using the Johnson-Neyman procedure, which indicates at which levels of a moderator an independent variable has a significant effect on the dependent variable [7]. Controlling for age and sex, the effect of diagnosis on TBV was significant for IQ scores less than 115.3 (ASD > TDC) and greater than 140.1 (TDC > ASD). This means that for IQ scores below 115 and above 140, the relationship between IQ and TBV differs between ASD and TDC. Within the TDC group, the semi-partial correlation of IQ with TBV given age and sex was *r* = 0.38, *p* < 0.001. Within the ASD group, this correlation was *r* = 0.045, *p* = 0.47. Thus, the typical positive correlation between IQ and TBV was absent in the ASD group.

Across both groups, age negatively predicted GMV, cortical GMV, and cerebellar GMV. Age positively predicted WMV, cortical WMV, and cerebellar WMV. Age did not significantly predict TBV. Notably, there were no significant age-by-diagnosis interactions in any of the models tested.

Sex was a significant predictor in every model, with large effects (male larger than female) on all measures except cerebellar WMV, on which it had a small effect. Notably, there were no significant sex-by-diagnosis interactions. There were significant main effects of IQ in all models except the cerebellar WMV. All significant IQ effects occurred in the presence of an IQ-by-diagnosis interaction.

Models were all tested including height as a predictor in the subset of individuals for whom there was an available measure of height within a year of the scan, with few changes to the significance of results. In white matter models (WMV, cortical WMV, and cerebellar WMV), age became non-significant as a predictor, likely due to the multicollinearity (age and height were highly correlated, *r* = 0.86, *p* < 0.001). Within the TBV model, age became a significant predictor (partial *η*^2^ =0.03, *p* < 0.01). The only other qualitative change was in the model of cerebellar WMV, in which the effects of sex and diagnosis became non-significant, likely due to less statistical power compared to the full sample (*n =* 456 versus *n* = 281).

### Ventricles

**Fig. 3:**
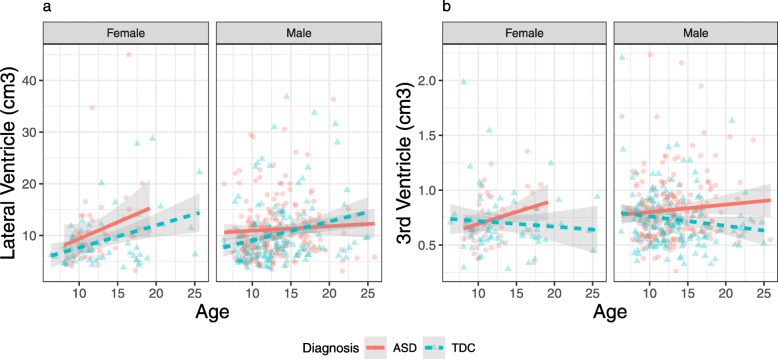
Ventricular volume in the primary sample

Controlling for age, sex, and IQ, diagnosis was a significant predictor of lateral ventricular volume (partial *η*^2^ = 0.013, *p* < 0.05). Visual inspection of data indicated one extreme outlier in lateral ventricular volume; removing this outlier reduced the size of the effect (partial *η*^2^ = 0.009, *p* < 0.05, Fig. 3). There was a significant age-by-diagnosis interaction in the third ventricles, (partial *η*^2^ = 0.013, *p* < 0.05). In the ventricles, unlike in the majority of the tissue volume measures, there were no significant IQ-by-diagnosis interactions in predicting volume.

### Gray matter-to-white matter ratio

To examine relative contributions of GMV and WMV differences between the groups, the ratio of GMV-to-WMV differences were examined in a model similar to those used to examine primary volumetric measures. This yielded no significant interaction terms, and no significant main effect of group or IQ. There were significant effects of age (partial *η*^2^ = 0.46, *p* < 0.001) and sex (partial *η*^2^ = 0.03, *p* < 0.05), with a greater gray-to-white ratio in females, and with this ratio decreasing with age (Figure S4, Additional file 1).

### Clinical correlates of brain size.

**Fig. 4:**
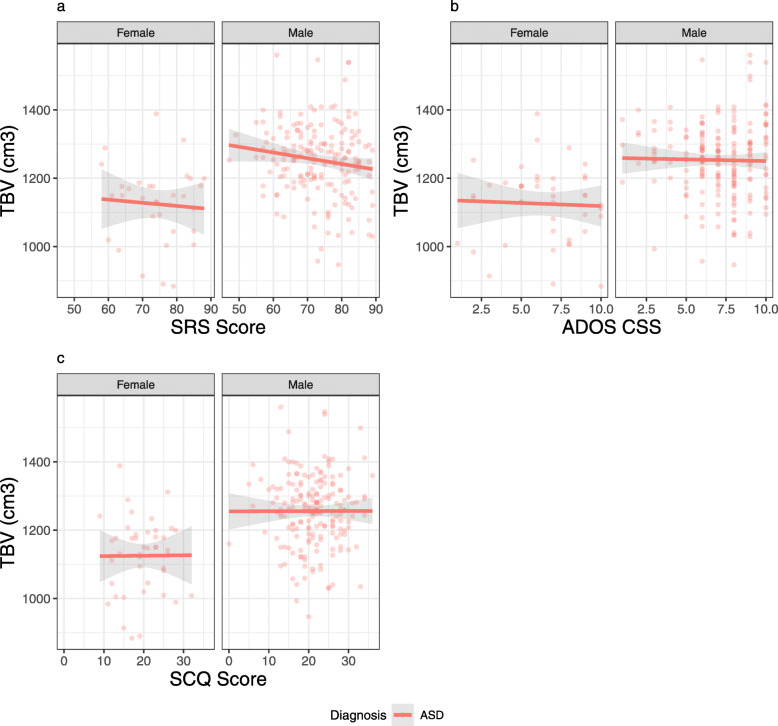
Relationships of clinical severity measures (**a**, SRS; **b**, ADOS CSS; **c**, SCQ) with TBV within the CHOP ASD group only. No severity measure showed a significant relationship with ASD symptoms, controlling for age, sex, and IQ

When controlling for age, sex, and IQ, neither the ADOS CSS, the SRS, nor the SCQ significantly predicted TBV within the ASD group (Fig. 4, Table 4). That is, none of the three measures of ASD severity correlated with brain volume in the ASD group.

**Table 4:** Models showing the relationships of clinical severity measures with TBV within the CHOP ASD group only. No severity measure showed a significant relationship with ASD symptoms, controlling for age, sex, and IQ

Clinical severity and total brain volume									
	SRS			ADOS CSS			SCQ		
	*B*	Std. Beta	*p*	*B*	Std. Beta	*p*	*B*	Std. Beta	*p*
(Intercept)	1202.68		< .001	1193.05		< .001	1165.85		< .001
IQ	0.3	0.05	0.382	0.28	0.05	0.417	0.39	0.07	0.252
Age	− 2.4	− 0.07	0.224	− 2.26	− 0.07	0.255	− 1.36	− 0.04	0.499
Sex	93.88	0.43	< .001	91.27	0.42	< .001	91.99	0.44	< .001
SRS	− 0.45	− 0.05	0.37						
ADOS CSS				− 0.43	− 0.01	0.893			
SCQ							0.17	0.01	0.883
Observations	234			238			233		
*R*^2^/adj. *R*^2^	.192/.178			.184/.170			.199/.185		

### Parental education

In order to obtain precise statistics accounting for the rank-order nature of the parental education data, zero-order correlations between parental education and brain volume within each group were examined with Kendall’s Tau. Within the TDC group, the relationship between TBV and parental education was significant and positive (τ = .21, *p* < 0.001, Figure S5, Additional file 1). This relationship was negative (although non-significant) within the ASD group (τ = − 0.09, *p* = 0.09). To explore the significance of this apparent disordinal interaction, parental education was added to the model of TBV, such that the full model was TBV ~ diagnosis + age + sex + IQ + parental education + IQ*diagnosis + parental education*diagnosis. In this model, the interaction of parental education and diagnosis was significant (partial *η*^2^ = 0.03, *p* < 0.01). This interaction is also significant in separate models in which father’s education is included as a binary factor (college degree or no college degree) indicating that this interaction effect is robust to choice of statistical method. When mother’s education is included as a binary factor, it is not significant (*p* = 0.12).

### Race

Because race was imbalanced between groups, all of the models in Table 2 were examined within only the White participants, to rule out the explanation that racial differences accounted for group differences. The significance of terms changed in only three models: in the model predicting cerebellar GMV, IQ, diagnosis, and the IQ-by-diagnosis interaction were no longer significant (possibly due to reduced power); in the model predicting cerebellar WMV, diagnosis was no longer significant; and in the model predicting third ventricle volume, sex and diagnosis became significant. Although diagnosis remained a significant predictor in all other models, the effect size of diagnosis was somewhat reduced.

### Extreme size subgroup analysis

To identify ASD participants with extremely large or small brains, we calculated the mean and standard deviation of TBV within 3-year age bins separately by sex within the TDC group, and examined ASD individuals whose TBV exceeded 2 SD from the mean for their age and sex. In the ASD group, there were 10 individuals with brains 2 SD above the mean for their age/sex bin (4.1%), and 10 with brains 2 SD below (4.1%, compared to 2.3% above and 2.8% below in the TDC group). Although a higher proportion of ASD individuals had brains with extreme sizes than TDC individuals, this difference was not statistically significant (*χ*^2^ (2, *N* = 456) = 1.9, *p* = 0.38). Information on the age, IQ, and gender ratio for the ASD individuals with larger, smaller, and typically-sized brains is presented in Table S3, Additional file 1. Comparing these groups statistically, there is a significant difference in age (*F* (2,237) = 3.95, *p* < 0.05), with the mean age of the extreme ASD groups higher than the mean age of the ASD individuals with typically sized brains. There were not significant differences in IQ (*F* (2, 237) = 0.12, *p* = 0.88), sex ratio (Fisher’s exact test *p* = 0.25), or ADOS CSS (*F* (2, 235) = 1.34, *p* = 0.26) between the groups. To investigate whether a subgroup of individuals with extremely sized brains drove the between-group diagnostic differences, the TBV model presented in Table 2 was re-examined excluding all ASD individuals with TBV > 2 SD from the mean, and the effect of diagnosis remained significant (partial *η*^2^ = 0.062, *p* < 0.001).

### Replication results

**Fig. 5:**
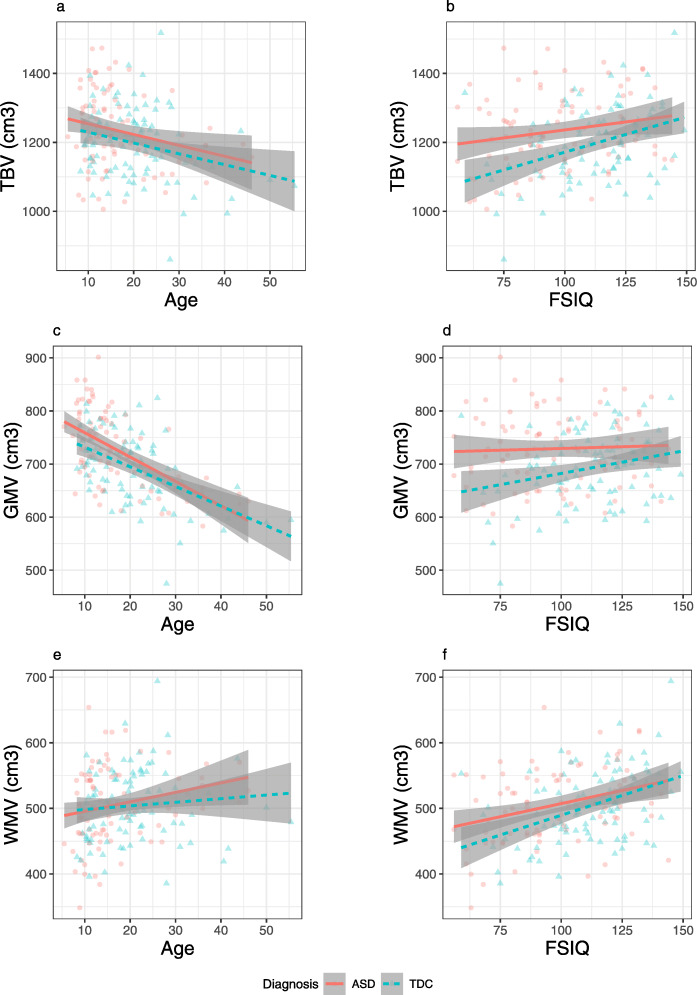
Relationships of IQ and age with total brain volume (**a**, **b**), gray matter volume (**c**, **d**), and white matter volume (**e**, **f**) in the Yale sample, by diagnosis. Age negatively predicted TBV and GMV (**a**, **c**). IQ positively predicted all three measures (**b**, **d**, **f**). ASD status positively predicted all three measures

As in the primary dataset, diagnosis was a significant predictor of TBV, GMV, and WMV in the Yale dataset (Tables 5 and 6, Fig. 5). There was no significant interaction of age and diagnosis in any of the models. There was also no significant interaction of diagnosis and IQ. These interactions were dropped from the models for simplicity, but are presented in Table S4, Additional file 1. In the full models with all interaction terms, the main effects of diagnosis on TBV, GMV, and WMV were in the same direction as in the main-effects-only models (ASD > TDC), but were not significant, potentially due to the loss of degrees of freedom. Although there was not a significant IQ-by-diagnosis interaction, the correlation between IQ and TBV was qualitatively smaller in the ASD sample than the TDC sample, which was the pattern observed in the primary dataset. Within the TDC group, the semi-partial correlation of IQ with TBV given age was *r* = 0.35, *p* < 0.001. Within the ASD group, this correlation was *r* = 0.25, *p* < 0.05. IQ was a significant predictor of TBV, GMV, and WMV. Age was a significant predictor of TBV and GMV. As in the primary dataset, diagnosis did not predict the ratio of GMV-to-WMV (Figure S6, Additional file 1). Additionally, both lateral ventricles and third ventricles were enlarged in the ASD group (Figure S7, Additional file 1). In the ASD subgroup analysis, there were 7 individuals with brains 2 SD above the mean for their age bin (9.1%), and 2 with brains 2 SD below (2.6%, compared to 2.4% above and 0% below in the TDC group). Fisher’s exact test indicates that the proportion of individuals with extremely-sized brains is different between the ASD and TDC groups (*p* = 0.035). Within the ASD group, there were no differences between the small, large, and typically-sized subgroups in age (*F* (2,74) = 0.49, *p* = 0.62) or IQ (*F* (2,74) = 0.33, *p* = 0.72). In models testing the main effects of IQ, age, and diagnosis on TBV when excluding the extremely-sized ASD individuals, diagnosis remained significant (partial *η*^2^ = 0.027, *p* = 0.04).

**Table 5:** Main effects of IQ, Age, and Diagnosis in the Yale sample.

Yale Effects of IQ, Age, and Diagnosis												
*Predictors*	*Estimates*	*std. Beta*	*p*	*partial η2*	*Estimates*	*std. Beta*	*p*	*partial η2*	*Estimates*	*std. Beta*	*p*	*partial η2*
	**TBV**				**GMV**				**WMV**			
Intercept	1142.35	0.21	**<0.001**	0.84	743.62	0.20	**<0.001**	0.88	399.25	0.16	**<0.001**	0.70
IQ	1.52	0.31	**<0.001**	0.09	0.57	0.18	**0.005**	0.05	0.95	0.38	**<0.001**	0.13
Age	-3.40	-0.29	**<0.001**	0.09	-4.23	-0.56	**<0.001**	0.32	0.82	0.13	0.064	0.02
Diagnosis	-46.16	-0.42	**0.006**	0.04	-27.85	-0.39	**0.003**	0.05	-18.36	-0.32	**0.035**	0.03
R^2^ / R^2^ adjusted	0.180 / 0.166				0.380 / 0.369				0.150 / 0.135			
	**Cortical GMV**				**Cortical WMV**							
Intercept	577.03	0.21	**<0.001**	0.86	376.56	0.16	**<0.001**	0.69				
IQ	0.40	0.15	**0.019**	0.03	0.89	0.37	**<0.001**	0.12				
Age	-3.67	-0.57	**<0.001**	0.34	0.77	0.13	0.073	0.02				
Diagnosis	-24.53	-0.41	**0.002**	0.06	-16.98	-0.31	**0.042**	0.02				
R^2^ / R^2^ adjusted	0.398 / 0.387				0.145 / 0.130							
	**Cerebellum**				**Cerebellar GMV**				**Cerebellar WMV**			
Intercept	130.65	0.11	**<0.001**	0.79	107.96	0.07	**<0.001**	0.80	22.69	0.19	**<0.001**	0.62
IQ	0.16	0.26	**0.001**	0.06	0.11	0.21	**0.007**	0.04	0.05	0.32	**<0.001**	0.09
Age	-0.29	-0.19	**0.010**	0.04	-0.35	-0.28	**<0.001**	0.08	0.06	0.14	0.059	0.02
Diagnosis	-2.94	-0.21	0.180	0.01	-1.55	-0.14	0.384	0.00	-1.38	-0.37	**0.019**	0.03
R^2^ / R^2^ adjusted	0.092 / 0.076				0.110 / 0.094				0.117 / 0.102			
	**Lateral Ventricles**				**Third Ventricles**				**GMV:WMV Ratio**			
Intercept	9.10	0.27	**0.001**	0.06	0.84	0.21	**<0.001**	0.30	1.78	0.02	**<0.001**	0.92
IQ	0.02	0.07	0.375	0.00	-0.00	-0.00	0.956	0.00	-0.00	-0.22	**<0.001**	0.08
Age	0.28	0.34	**<0.001**	0.11	0.01	0.22	**0.005**	0.05	-0.01	-0.66	**<0.001**	0.47
Diagnosis	-4.19	-0.54	**<0.001**	0.07	-0.11	-0.41	**0.011**	0.04	-0.01	-0.04	0.698	0.00
R^2^ / R^2^ adjusted	0.148 / 0.133				0.070 / 0.053				0.529 / 0.521

**Table 6:** Mean and standard deviation of volumes in each group in the Yale sample, and Cohen’s *d* for the difference between groups. Note that groups are not matched for age and IQ, and that Cohen’s *d* does not account for these factors

Yale group means			
	ASD mean (SD)	TDC mean (SD)	Cohen’s *d*
TBV	1233.38 (110.9)	1196.13 (108.09)	0.34
GMV	728.94 (71.39)	692.51 (65.65)	0.53
WMV	504.96 (58.38)	504.06 (55.87)	0.02
Cortical GMV	554.87 (60.33)	521.45 (55.1)	0.58
Cortical WMV	476.16 (55.99)	475.63 (53.6)	0.01
Cerebellum	141.32 (14.18)	139.58 (13.73)	0.12
Cerebellar GMV	112.52 (11.76)	111.15 (11.24)	0.12
Cerebellar WMV	28.79 (4.03)	28.43 (3.53)	0.1
Lateral ventricles	15.99 (8.58)	13.29 (6.65)	0.36
Third ventricle	0.94 (0.29)	0.86 (0.23)	0.32

## Discussion

We do not find evidence to support the prediction that brain size in ASD normalizes over development. The Early Overgrowth hypothesis predicts that either (1) there should be no significant main effect of diagnosis on brain size (i.e., that brain size has normalized between the groups aged 6–25 years old in our sample) or (2) there should be a significant interaction of age and diagnosis, with volumetric differences for the youngest autistic children and normalization of brain volume across the age range. Our findings do not support either prediction. In the primary sample, we found a significant main effect of diagnosis for GMV, WMV, and TBV, and no significant interaction with age, with volumes about 2.8–3.2% larger in the ASD group. This finding was replicated in the Yale sample, in which TBV and GMV are 3.1% and 5.3% larger in the ASD group, with no interactions with age. Furthermore, our explorations of sub-groups of ASD individuals with particularly enlarged brains (> 2 SD from the typical mean for their age/sex) suggested that this enlargement was slightly more common in older youth in the CHOP sample and consistent across ages in the Yale sample. This indicates that findings of group-level enlargement in ASD were not driven by a subset of only the youngest children in our samples having enlarged brains. In addition, we observed a significant interaction in the primary sample between diagnosis and IQ, such that the overall brain enlargement effect in ASD was driven by children with IQ scores less than 115. This interaction is due to the stronger correlation in the TDC sample between IQ and brain volume than in the ASD sample, which showed no relationship between IQ and brain volume. The main effect of diagnosis should be interpreted in light of this significant interaction with IQ. Nevertheless, this study finds converging evidence in two large datasets that early brain overgrowth persists through adolescence and into early adulthood in ASD, failing to support “normalization” predictions from the Early Overgrowth hypothesis.

Increased brain volume has been one of the most consistently observed biomarkers of ASD in young children. Our results suggest that brain enlargement persists into early adulthood. This is consistent with some prior publications, including MRI studies [32, 33, 57, 61, 76] and a very large study of head circumference [17], but not with others [3, 35, 37, 52, 63, 70, 80]. The current results are noteworthy because of the large samples collected on the same scanner (by sample), inclusion of a broad age and IQ range, and large female representation. The size and quality of the samples we report allow for more generalizable and definitive conclusions about the development of brain size in autism than have previously been possible from smaller studies.

These results support neither the model of GM/WM imbalance predicting increased GM but decreased WM in ASD [12], nor the model predicting greater effect sizes of diagnostic group on WM than GM [41]. Rather, the data suggest that structural differences in WM occur in roughly equal proportion to GM between groups.

There are several potential mechanisms underlying the persistent brain volume difference in ASD. Brain volume is the product of cortical thickness and cortical surface area, which are independently heritable and have unique mechanistic underpinnings [58]. Increased surface area has been identified in some ASD samples [56] but not others [62]. Greater cortical thickness or differential rates of change have also been observed in some studies [25, 46, 62, 73]. but not all [56]. Using a subset of the CHOP sample reported in the current study, we recently reported that regional deviations from a normative model of brain development in diffusion metrics, volume, thickness, and surface area can accurately classify diagnostic status, although diffusion metrics out-performed anatomical measures in this age-based approach [82]. We plan to further investigate regional differences in cortical surface area and thickness in future work.

In typical development, dendritic arborization and synaptogenesis occur rapidly in the first year of life, followed by dendritic pruning [42]. The emergence of brain volume differences and clinical symptoms across the first 2 years of life in ASD points to these as candidate mechanisms. One potential mechanism of reduced dendritic pruning is mammalian target of rapamycin (mTOR) kinase, which is regulated by a number of genes associated with ASD, including *TSC1*/*TSC2*, *NF1*, and *PTEN* [13]. Hyperactive mTOR can produce excessive synaptic proteins and impair autophagy, and has been correlated with increased dendritic spine density in post-mortem brains of autistic individuals [79]. Another potential genetic source of this effect is chromodomain helicase DNA binding protein 8 (*CHD8*). This regulatory gene with neurodevelopmental targets has been strongly associated with ASD [69], and has been clinically associated with macrocephaly in ASD and in zebrafish models [9]. These cellular processes may be expressed differentially in different regions of the brain. For example, post-mortem studies of neuronal density revealed higher density in some regions of autistic brains compared to controls [16], but lower density in other regions [84].

In the context of significantly larger brains on average in ASD, we find no correlation between any of our severity measures and TBV, consistent with prior findings in preschoolers [1]. The failure to find correlations with symptom severity complicates the clinical implications of the enlarged-brain biomarker, given the conceptualization of autism as a spectrum disorder. If the degree of brain enlargement is not associated with the degree of core ASD symptoms, it is unclear how increased brain volume is functionally important to causal mechanisms of ASD. It might be that increased brain volume is not an underlying source of core ASD symptom differences, but represents a collateral consequence of the true underlying source. If true, increased brain size could be a biomarker of ASD, without being central to the pathophysiology [77]. Alternatively, enlarged brain volume may represent a categorical diathesis for ASD, with dimensional causes and symptoms overlaid. Another alternative is that global volume differences may not entirely reflect localized differences in regions, pathways, and networks, which may correlate more closely with symptom severity. Another important alternative is that group-level volume differences may be driven by a subsample of individuals with both ASD and enlarged brains, who are not distinguished by clinical severity [1]. We find that 4.1% of the CHOP ASD group and 9.1% of the Yale ASD group had brain volume greater than 2 SD above the mean for their age and sex. However, neither IQ nor clinical severity differed between the subgroups with extremely large or small brains and the subgroup falling within the typical range. Importantly, the group-average difference in brain volume remains significant when excluding the autistic individuals with extremely-sized brains, indicating that group level differences are not entirely driven by a subgroup of individuals. Finally, the absence of a correlation between brain size and ASD severity might indicate that our ASD symptom metrics fail to capture important aspects of ASD heterogeneity, as our three measures are poorly correlated with one another in this sample and in others [11].

Diagnostic group differences in brain volume are also complicated by an interaction between diagnosis and IQ. In humans, the relationship between brain size and intelligence has long been noted [53, 60, 91]. Indeed, in both the primary and replication datasets, we find a significant correlation within the TDC sample between brain size and IQ, while correcting for sex and age. However, correlations within the ASD group are smaller, and for the primary sample are not significant. The lack of a correlation with IQ suggests that individual differences in brain size have a different meaning in ASD, and that additional tissue volume does not confer cognitive advantages.

What, mechanistically, might disrupt the relationship between brain volume and IQ in ASD? The relationship may be weakened through a combination of underlying, unmeasured microstructural differences or differences in network organization. Alternatively, if IQ measurement is less reliable in ASD than TDC, the correlation between IQ and brain volume would be attenuated. While the IQ measures used in this study have evidence of validity and reliability in both typical and clinical [26] and ASD-specific samples [90], evidence of test-retest reliability in an ASD sample is lacking, and the factor structure of IQ may be different in ASD [18].

Our regions-of-significance analyses suggest that the ability of a study to detect brain enlargement in ASD depends on the IQ of the TDC sample. We expect little between-group difference when TDCs have high IQs, and greater difference when TDCs have lower IQs. Even a sample well-matched on IQ would be expected to show little difference if both groups are high in IQ. Failing to include lower-IQ TDC participants in imaging studies may bias results toward null brain volume differences between ASD and TDC, and contribute to controversy over the persistence of brain enlargement in ASD. Although the IQ-by-diagnosis interaction was not significant in the Yale sample, there are several reasons to believe the CHOP dataset is superior in accuracy and sensitivity (i.e., greater sample size, 3T versus 1.5T scanner, improved scan sequences, diversity of sex, superior matching of demographics). Post hoc power analyses using the effect sizes of the interaction obtained in the CHOP sample indicate that the power to detect this interaction in the Yale sample was 0.53 for TBV, 0.65 for GMV, and 0.29 for WMV. Thus, even the relatively large Yale dataset was likely underpowered to detect these interactions.

Although expected sex-effects were observed (i.e., larger brains in males than females), no sex-by-diagnosis interactions were observed in any of the measures. These findings suggest that diagnostic group differences in global brain morphology are not related to sex.

In the TDC sample, higher parental educational attainment (a proxy for socioeconomic status, SES) was associated with increased TBV and higher child IQ. These findings are both consistent with a theoretical model of brain structure mediating the relationship between parent SES and child cognitive ability [55]. Interestingly, follow-up analyses found that the relationship between parental education and child’s IQ is attenuated in the ASD sample, and that the relationship between parental education and child’s brain volume is weak and reversed in ASD. These findings suggest that the mechanisms that result in enlarged brains in ASD disrupt the typical relationship between SES and neurocognitive development, as well as the relationship between brain size and cognitive ability. Additionally, including the interaction of parental education-by-diagnosis in a regression model predicting TBV increases the partial *η2* value of the main effect of diagnosis (from 0.05 to 0.11). This finding highlights the importance of obtaining information about cognitive ability and educational attainment of parents. Such information allows for the study of not only the autistic individual’s ability, but also how much that ability deviates from predicted familial relationships in the absence of ASD.

## Limitations

Our samples’ age range (6–25 years) is a significant limitation to our ability to fully evaluate the Early Brain Overgrowth hypothesis. Normalization is proposed to occur immediately following the period of overgrowth [20], with brain sizes of ASD and TDC equalizing by approximately age 5. Therefore, it is possible that the magnitude of the group differences observed in our sample would have been larger had they been observed as toddlers. A second limitation is that our data are cross sectional. This would be most problematic if there is a systematic difference in the brain volumes of individuals who chose to participate at different ages. The ideal test of the Early Brain Overgrowth hypothesis would follow a cohort prospectively from diagnosis as a toddler to adulthood. Longitudinal study is particularly important to assess individual differences in growth trajectories. For example, it is possible that a subset of our sample had larger brains relative to peers as toddlers, experienced normalization, and now have average-sized brains, while other individuals’ brain size did not normalize and remained enlarged. The group differences we report demonstrate clearly that brain volume changes do persist at a group level in autistic adolescents and adults, and further longitudinal study should investigate the potential clinical implications of differing individual trajectories.

## Conclusions

In summary, this work provides evidence that brain volume does not normalize by school-age, adolescence, or young-adulthood in ASD. While the effect sizes obtained in both samples are somewhat smaller than those often reported in samples of toddlers, enlargement remains. As we do not have MRIs from younger ages, it is possible that some degree of normalization occurred prior to the present measurements; if so, this normalization was not exhaustive. It is important that cellular and molecular researchers understand this developmental context in the search for mechanisms that might account for brain overgrowth in ASD.

## Supplementary information

**Additional file 1: Table S1.** Height, race, and parental education for the primary sample. Fisher’s Exact Test was used to test for differences in proportion of race due to small sizes within cells. **Figure S1.** Age and IQ distributions in the primary sample. **Figure S2.** Age and IQ distributions in the replication sample. **Figure S3.** Volume of cerebellar white matter in original and reprocessed images in the primary sample. Low reliability of this measure is clearly driven by one subject. **Figure S4.** Ratio of gray to white matter in the primary sample. Table S2 part 1. CHOP models with effects of IQ, Age, Sex, Diagnosis, IQ*Diagnosis, Age*Diagnosis, Sex*Diagnosis. Table S2 part 2. CHOP models with effects of IQ, Age, Sex, Diagnosis, IQ*Diagnosis, Age*Diagnosis, Sex*Diagnosis. **Figure S5.** Relationships of parental education with (a) brain volume and (b) IQ, in the subset of the CHOP sample for which parental education was available. Within the TDC sample, a positive relationship was observed between parent education and both TBV and IQ. Within the ASD group, the positive relationship between parent education and IQ was attenuated, and the relationship with TBV was reversed. **Table S4.** Yale models with effects of IQ, Age, Diagnosis, IQ*Diagnosis, Age*Diagnosis.

## Data Availability

The data that support the findings of this study are available from the corresponding author, upon reasonable request.

## Abbreviations

ASD: Autism spectrum disorder
GMV: Gray matter volume
TBV: Total brain volume
TDC: Typically developing control
WMV: White matter volume

## Footnotes

1 We use identity-first language per our stakeholder preference [45]

## References

[1] Amaral DG, Li D, Libero L, Solomon M, Van de Water J, Mastergeorge A, Naigles L, Rogers S, Wu Nordahl C. In pursuit of neurophenotypes: The consequences of having autism and a big brain. Autism Res. 2017. 10.1002/aur.1755. 10.1002/aur.1755 PMC5520638 28239961

[2] Auzias, G., Breuil, C., Takerkart, S., & Deruelle, C. (2014). Detectability of brain structure abnormalities related to autism through MRI-derived measures from multiple scanners. 314–317. 10.1109/BHI.2014.6864366.

[3] Aylward EH, Minshew NJ, Field K, Sparks BF, Singh N (2002). Effects of age on brain volume and head circumference in autism. Neurology.

[4] Bailey A, Le Couteur A, Gottesman I, Bolton P, Simonoff E, Yuzda E, Rutter M (1995). Autism as a strongly genetic disorder: Evidence from a British twin study. Psycholog Med.

[5] Baio J. Prevalence of Autism Spectrum Disorder Among Children Aged 8 Years—Autism and Developmental Disabilities Monitoring Network, 11 Sites, United States, 2014. MMWR. Surveillance Summaries. 2018:67. 10.15585/mmwr.ss6706a1. 10.15585/mmwr.ss6706a1 PMC5919599 29701730

[6] Bartholomeusz HH, Courchesne E, Karns CM (2002). Relationship Between Head Circumference and Brain Volume in Healthy Normal Toddlers, Children, and Adults. Neuropediatrics.

[7] Bauer DJ, Curran PJ (2005). Probing Interactions in Fixed and Multilevel Regression: Inferential and Graphical Techniques. Multivariate Behavioral Research.

[8] Beacher FD, Minati L, Baron-Cohen S, Lombardo MV, Lai M-C, Gray MA, Harrison NA, Critchley HD (2012). Autism Attenuates Sex Differences in Brain Structure: A Combined Voxel-Based Morphometry and Diffusion Tensor Imaging Study. Am J Neuroradiol.

[9] Bernier R, Golzio C, Xiong B, Stessman HA, Coe BP, Penn O, Witherspoon K, Gerdts J, Baker C, Vulto-van Silfhout AT, Schuurs-Hoeijmakers JH, Fichera M, Bosco P, Buono S, Alberti A, Failla P, Peeters H, Steyaert J, Vissers LELM (2014). Disruptive CHD8 mutations define a subtype of autism early in development. Cell.

[10] Bloss CS, Courchesne E (2007). MRI Neuroanatomy in Young Girls With Autism: A Preliminary Study. J Am Acad Child Adolesc Psychiatry.

[11] Bölte S, Westerwald E, Holtmann M, Freitag C, Poustka F (2011). Autistic Traits and Autism Spectrum Disorders: The Clinical Validity of Two Measures Presuming a Continuum of Social Communication Skills. J Autism Dev Disord.

[12] Bonilha L, Cendes F, Rorden C, Eckert M, Dalgalarrondo P, Li LM, Steiner CE (2008). Gray and white matter imbalance – Typical structural abnormality underlying classic autism?. Brain Dev.

[13] Bourgeron T (2009). A synaptic trek to autism. Curr Opin Neurobiol.

[14] Button KS, Ioannidis JPA, Mokrysz C, Nosek BA, Flint J, Robinson ESJ, Munafò MR (2013). Power failure: Why small sample size undermines the reliability of neuroscience. Nat Rev Neurosci.

[15] Carper RA, Moses P, Tigue ZD, Courchesne E (2002). Cerebral lobes in autism: Early hyperplasia and abnormal age effects. Neuro Image.

[16] Casanova MF, van Kooten IAJ, Switala AE, van Engeland H, Heinsen H, Steinbusch HWM, Hof PR, Trippe J, Stone J, Schmitz C (2006). Minicolumnar abnormalities in autism. Acta Neuropathologica.

[17] Chaste P, Klei L, Sanders SJ, Murtha MT, Hus V, Lowe JK, Willsey AJ, Moreno-De-Luca D, Yu TW, Fombonne E, Geschwind D, Grice DE, Ledbetter DH, Lord C, Mane SM, Lese Martin C, Martin DM, Morrow EM, Walsh CA (2013). Adjusting head circumference for covariates in autism: Clinical correlates of a highly heritable continuous trait. Biological Psychiatry.

[18] Clements CC, Watkins MW, Schultz RT, Yerys BE. Does the Factor Structure of IQ Differ Between the Differential Ability Scales (DAS-II) Normative Sample and Autistic Children? Autism Research, n/a(n/a). 2020. 10.1002/aur.2285. 10.1002/aur.2285 32112626

[19] Constantino, J., & Gruber, C. (2012). *The Social Responsiveness Scale Manual, Second Edition (SRS-2)*. Western Psychological Services.

[20] Courchesne E (2004). Brain development in autism: Early overgrowth followed by premature arrest of growth. Ment Retard Dev Disabil Res Rev.

[21] Courchesne E, Carper R, Akshoomoff N (2003). Evidence of brain overgrowth in the first year of life in autism. JAMA.

[22] Courchesne E, Karns CM, Davis HR, Ziccardi R, Carper RA, Tigue ZD, Chisum HJ, Moses P, Pierce K, Lord C, Lincoln AJ, Pizzo S, Schreibman L, Haas RH, Akshoomoff NA, Courchesne RY (2001). Unusual brain growth patterns in early life in patients with autistic disorder: An MRI study. Neurology.

[23] Dale AM, Fischl B, Sereno MI (1999). Cortical Surface-Based Analysis: I. Segmentation and Surface Reconstruction. Neuro Image.

[24] Di Martino A, Yan C-G, Li Q, Denio E, Castellanos FX, Alaerts K, Anderson JS, Assaf M, Bookheimer SY, Dapretto M, Deen B, Delmonte S, Dinstein I, Ertl-Wagner B, Fair DA, Gallagher L, Kennedy DP, Keown CL, Keysers C (2014). The autism brain imaging data exchange: Towards a large-scale evaluation of the intrinsic brain architecture in autism. Mol Psychiatry.

[25] Ecker C, Shahidiani A, Feng Y, Daly E, Murphy C, D’Almeida V, Deoni S, Williams SC, Gillan N, Gudbrandsen M, Wichers R, Andrews D, Van Hemert L, Murphy DGM (2014). The effect of age, diagnosis, and their interaction on vertex-based measures of cortical thickness and surface area in autism spectrum disorder. J Neural Transm.

[26] Elliot, C. (2007). The Differential Abilities Scale, Second Edition. Harcourt Assessments, Inc.

[27] Fischl B, Dale AM (2000). Measuring the thickness of the human cerebral cortex from magnetic resonance images. Proc Natl Acad Sci.

[28] Fischl B, Salat DH, Busa E, Albert M, Dieterich M, Haselgrove C, van der Kouwe A, Killiany R, Kennedy D, Klaveness S, Montillo A, Makris N, Rosen B, Dale AM (2002). Whole Brain Segmentation: Automated Labeling of Neuroanatomical Structures in the Human Brain. Neuron.

[29] Fischl B, Salat DH, van der Kouwe AJW, Makris N, Ségonne F, Quinn BT, Dale AM (2004). Sequence-independent segmentation of magnetic resonance images. Neuro Image.

[30] Fombonne E, Rogé B, Claverie J, Courty S, Frémolle J (1999). Microcephaly and macrocephaly in autism. J Autism Dev Disord.

[31] Fortin J-P, Parker D, Tunç B, Watanabe T, Elliott MA, Ruparel K, Roalf DR, Satterthwaite TD, Gur RC, Gur RE, Schultz RT, Verma R, Shinohara RT (2017). Harmonization of multi-site diffusion tensor imaging data. Neuro Image.

[32] Freitag CM, Luders E, Hulst HE, Narr KL, Thompson PM, Toga AW, Krick C, Konrad C (2009). Total Brain Volume and Corpus Callosum Size in Medication-Naïve Adolescents and Young Adults with Autism Spectrum Disorder. Biol Psychiatry.

[33] Goldman S, Wang C, Salgado MW, Greene PE, Kim M, Rapin I (2009). Motor stereotypies in children with autism and other developmental disorders. Dev Med Child Neurol.

[34] Gotham K, Pickles A, Lord C (2009). Standardizing ADOS Scores for a Measure of Severity in Autism Spectrum Disorders. J Autism Dev Disord.

[35] Hallahan B, Daly EM, McAlonan G, Loth E, Toal F, FO B, Robertson D, Hales S, Murphy C, Murphy KC, Murphy DGM (2009). Brain morphometry volume in autistic spectrum disorder: A magnetic resonance imaging study of adults. Psychol Med.

[36] Hardan AY, Minshew NJ, Mallikarjuhn M, Keshavan MS (2001). Brain Volume in Autism. J Child Neurol.

[37] Hardan AY, Muddasani S, Vemulapalli M, Keshavan MS, Minshew NJ (2006). An MRI Study of Increased Cortical Thickness in Autism. Am J Psychiatry.

[38] Hazlett HC, Gu H, McKinstry RC, Shaw DWW, Botteron KN, Dager SR, Styner M, Vachet C, Gerig G, Paterson SJ, Schultz RT, Estes AM, Evans AC, Piven J, Network IBIS (2012). Brain volume findings in 6-month-old infants at high familial risk for autism. Am J Psychiatry.

[39] Hazlett HC, Gu H, Munsell BC, Kim SH, Styner M, Wolff JJ, Elison JT, Swanson MR, Zhu H, Botteron KN, Collins DL, Constantino JN, Dager SR, Estes AM, Evans AC, Fonov VS, Gerig G, Kostopoulos P, McKinstry RC (2017). Early brain development in infants at high risk for autism spectrum disorder. Nature.

[40] Hazlett HC, Poe M, Gerig G, Smith RG, Provenzale J, Ross A, Gilmore J, Piven J (2005). Magnetic Resonance Imaging and Head Circumference Study of Brain Size in Autism: Birth Through Age 2 Years. Arch Gen Psychiatry.

[41] Herbert MR, Ziegler DA, Makris N, Filipek PA, Kemper TL, Normandin JJ, Sanders HA, Kennedy DN, Caviness VS (2004). Localization of white matter volume increase in autism and developmental language disorder. Ann Neurol.

[42] Huttenlocher PR (1990). Morphometric study of human cerebral cortex development. Neuropsychologia.

[43] Kanner L (1943). Autistic disturbances of affective contact. Neuro Child.

[44] Katuwal GJ, Baum SA, Cahill ND, Dougherty CC, Evans E, Evans DW, Moore GJ, Michael AM. Inter-Method Discrepancies in Brain Volume Estimation May Drive Inconsistent Findings in Autism. Front Neurosci. 2016;10. 10.3389/fnins.2016.00439. 10.3389/fnins.2016.00439 PMC5043189 27746713

[45] Kenny L, Hattersley C, Molins B, Buckley C, Povey C, Pellicano E (2016). Which terms should be used to describe autism? Perspectives from the UK autism community. Autism.

[46] Khundrakpam BS, Lewis JD, Kostopoulos P, Carbonell F, Evans AC (2017). Cortical Thickness Abnormalities in Autism Spectrum Disorders Through Late Childhood, Adolescence, and Adulthood: A Large-Scale MRI Study. Cerebral Cortex.

[47] Kvarven A, Strømland E, Johannesson M. Comparing meta-analyses and preregistered multiple-laboratory replication projects. Nature Human Behaviour, 1–12. 2019. 10.1038/s41562-019-0787-z. 10.1038/s41562-019-0787-z 31873200

[48] Lin L (2018). Bias caused by sampling error in meta-analysis with small sample sizes. PloS One.

[49] Lindley AA, Benson JE, Grimes C, Cole TM, Herman AA (1999). The relationship in neonates between clinically measured head circumference and brain volume estimated from head CT-scans. Early Hum Dev.

[50] Lord C, Risi S, Lambrecht L, Cook EH, Leventhal BL, DiLavore PC, Pickles A, Rutter M. The autism diagnostic observation schedule-generic: A standard measure of social and communication deficits associated with the spectrum of autism. J Autism Dev Disord. 2000;30. 10.1023/A:1005592401947. 11055457

[51] Masi A, DeMayo MM, Glozier N, Guastella AJ (2017). An Overview of Autism Spectrum Disorder, Heterogeneity and Treatment Options. NeurosciBull.

[52] McAlonan GM, Cheung V, Cheung C, Suckling J, Lam GY, Tai KS, Yip L, Murphy DGM, Chua SE (2005). Mapping the brain in autism. A voxel-based MRI study of volumetric differences and intercorrelations in autism. Brain.

[53] McDaniel MA (2005). Big-brained people are smarter: A meta-analysis of the relationship between in vivo brain volume and intelligence. Intelligence.

[54] Movsas TZ, Pinto-Martin JA, Whitaker AH, Feldman JF, Lorenz JM, Korzeniewski SJ, Levy SE, Paneth N (2013). Autism Spectrum Disorder is associated with ventricular enlargement in a Low Birth Weight Population. J Pediatr.

[55] Noble KG, Houston SM, Brito NH, Bartsch H, Kan E, Kuperman JM, Akshoomoff N, Amaral DG, Bloss CS, Libiger O, Schork NJ, Murray SS, Casey BJ, Chang L, Ernst TM, Frazier JA, Gruen JR, Kennedy DN, Van Zijl P (2015). Family income, parental education and brain structure in children and adolescents. Nat Neurosci.

[56] Ohta H, Nordahl CW, Iosif A-M, Lee A, Rogers S, Amaral DG (2016). Increased Surface Area, but not Cortical Thickness, in a Subset of Young Boys With Autism Spectrum Disorder: Cortical thickness in autism spectrum disorder. Autism Res.

[57] Palmen SJMC, Pol HEH, Kemner C, Schnack HG, Janssen J, Kahn RS, van Engeland H (2004). Larger Brains in Medication Naive High-Functioning Subjects with Pervasive Developmental Disorder. J Autism Dev Disord.

[58] Panizzon MS, Fennema-Notestine C, Eyler LT, Jernigan TL, Prom-Wormley E, Neale M, Jacobson K, Lyons MJ, Grant MD, Franz CE, Xian H, Tsuang M, Fischl B, Seidman L, Dale A, Kremen WS (2009). Distinct Genetic Influences on Cortical Surface Area and Cortical Thickness. Cerebral Cortex (New York, NY).

[59] Papademetris X, Jackowski MP, Rajeevan N, DiStasio M, Okuda H, Constable RT, Staib LH (2006). BioImage Suite: An integrated medical image analysis suite: An update. Insight J.

[60] Pietschnig J, Penke L, Wicherts JM, Zeiler M, Voracek M (2015). Meta-analysis of associations between human brain volume and intelligence differences: How strong are they and what do they mean?. Neuroscience & Biobehavioral Reviews.

[61] Piven J, Arndt S, Bailey J, Havercamp S, Andreasen N, Palmer P (1995). An MRI study of brain size in autism. Am J Psychiatry.

[62] Raznahan A, Lenroot R, Thurm A, Gozzi M, Hanley A, Spence SJ, Swedo SE, Giedd JN (2013). Mapping cortical anatomy in preschool aged children with autism using surface-based morphometry. NeuroImage Clin.

[63] Redcay E, Courchesne E (2005). When Is the Brain Enlarged in Autism? A Meta-Analysis of All Brain Size Reports. Biol Psychiatry.

[64] Reiss AL, Abrams MT, Singer HS, Ross JL, Denckla MB (1996). Brain development, gender and IQ in children. A volumetric imaging study. Brain: A Journal of Neurology.

[65] Richardson JTE (2011). Eta squared and partial eta squared as measures of effect size in educational research. Educ Res Rev.

[66] Rutter, M., Bailey, A., Lord, C., & et al. (2003). Social Communication Questionnaire, 2003. Western Psychological Services.

[67] Rutter M, Le Couteur A, Lord C, Faggioli R (2005). ADI-R: Autism diagnostic interview—Revised: Manual.

[68] Sacco R, Gabriele S, Persico AM (2015). Head circumference and brain size in autism spectrum disorder: A systematic review and meta-analysis. Psychiatry Research: Neuroimaging.

[69] Sanders SJ (2015). First glimpses of the neurobiology of autism spectrum disorder. Curr Opin Genet Dev.

[70] Scheel C, Rotarska-Jagiela A, Schilbach L, Lehnhardt FG, Krug B, Vogeley K, Tepest R (2011). Imaging derived cortical thickness reduction in high-functioning autism: Key regions and temporal slope. NeuroImage.

[71] Shi F, Wang L, Dai Y, Gilmore JH, Lin W, Shen D (2012). LABEL: Pediatric brain extraction using learning-based meta-algorithm. NeuroImage.

[72] Simonoff E, Pickles A, Charman T, Chandler S, Loucas T, Baird G (2008). Psychiatric Disorders in Children With Autism Spectrum Disorders: Prevalence, Comorbidity, and Associated Factors in a Population-Derived Sample. J Am Acad Child Adolesc Psychiatry.

[73] Smith E, Thurm A, Greenstein D, Farmer C, Swedo S, Giedd J, Raznahan A (2016). Cortical thickness change in autism during early childhood. Human Brain Mapping.

[74] Smith SM (2002). Fast robust automated brain extraction. Human Brain Mapping.

[75] Sparks BF, Friedman SD, Shaw DW, Aylward EH, Echelard D, Artru AA, Maravilla KR, Giedd JN, Munson J, Dawson G, Dager SR (2002). Brain structural abnormalities in young children with autism spectrum disorder. Neurology.

[76] Stanfield AC, McIntosh AM, Spencer MD, Philip R, Gaur S, Lawrie SM (2008). Towards a neuroanatomy of autism: A systematic review and meta-analysis of structural magnetic resonance imaging studies. European Psychiatry: The Journal of the Association of European Psychiatrists.

[77] Strimbu K, Tavel JA (2010). What are Biomarkers?. Curr Opin HIV AIDS.

[78] Styner, M. A., Charles, H. C., Park, J., & Gerig, G. (2002). Multisite validation of image analysis methods: Assessing intra- and intersite variability. 4684, 278–286. 10.1117/12.467167.

[79] Tang G, Gudsnuk K, Kuo S-H, Cotrina ML, Rosoklija G, Sosunov A, Sonders MS, Kanter E, Castagna C, Yamamoto A, Yue Z, Arancio O, Peterson BS, Champagne F, Dwork AJ, Goldman J, Sulzer D (2014). Loss of mTOR-Dependent Macroautophagy Causes Autistic-like Synaptic Pruning Deficits. Neuron.

[80] Tate DF, Bigler ED, McMahon W, Lainhart J (2007). The Relative Contributions of Brain, Cerebrospinal Fluid-Filled Structures and Non-Neural Tissue Volumes to Occipital-Frontal Head Circumference in Subjects with Autism. Neuropediatrics.

[81] Tick B, Bolton P, Happé F, Rutter M, Rijsdijk F (2016). Heritability of autism spectrum disorders: A meta-analysis of twin studies. J Child Psychol Psychiatry.

[82] Tunç B, Yankowitz LD, Parker D, Alappatt JA, Pandey J, Schultz RT, Verma R (2019). Deviation from normative brain development is associated with symptom severity in autism spectrum disorder. Mol Autism.

[83] Tustison NJ, Avants BB, Cook PA, Zheng Y, Egan A, Yushkevich PA, Gee JC (2010). N4ITK: Improved N3 bias correction. IEEE Trans Med Imaging.

[84] van Kooten IAJ, Palmen SJMC, von Cappeln P, Steinbusch HWM, Korr H, Heinsen H, Hof PR, van Engeland H, Schmitz C (2008). Neurons in the fusiform gyrus are fewer and smaller in autism. Brain.

[85] Wechsler, D. (1981). Manual for the Wechsler Adult Intelligence Scale—Revised. Psychological Corporation.

[86] Wechsler, D. (1991). Wechsler Intelligence Scale for Children: Third Edition manual. Psychological Corporation.

[87] Wechsler, D. (1997). Wechsler Adult Intelligence Scale–Third Edition. The Psychological Corporation.

[88] Wechsler, D. (1999). Wechsler Abbreviated Scale of Intelligence. The Psychological Corporation: Harcourt Brace & Company.

[89] Wechsler, D. (2011). Wechsler Abbreviated Scale of Intelligence–Second Edition (WASI-II). NCS Pearson.

[90] Wechsler, D., Kaplan, E., Fein, D., Kramer, J., Morris, R., Delis, D., & Maelender, A. (2003). Wechsler intelligence scale for children: Fourth edition (WISC-IV). Pearson.

[91] Willerman L, Schultz R, Rutledge N, Bigler E (1991). In vivo brain size and intelligence. Intelligence.

[92] Ypma RJF, Moseley RL, Holt RJ, Rughooputh N, Floris DL, Chura LR, Spencer MD, Baron-Cohen S, Suckling J, Bullmore ET, Rubinov M (2016). Default Mode Hypoconnectivity Underlies a Sex-Related Autism Spectrum. Biological Psychiatry: Cognitive Neuroscience and Neuroimaging.

[93] Yushkevich PA, Piven J, Hazlett HC, Smith RG, Ho S, Gee JC, Gerig G (2006). User-guided 3D active contour segmentation of anatomical structures: Significantly improved efficiency and reliability. NeuroImage.

