# Supplementary material for: Evidence against the “normalization” prediction of the early brain overgrowth hypothesis of autism
Source: Mol Autism. 2020 Jun 18;11:51. doi: 10.1186/s13229-020-00353-2 (PMC7301552; doi:10.1186/s13229-020-00353-2)
Supplement: Supplementary file 1 — Additional file 1: Table S1. Height, race, and parental education for the primary sample. Fisher’s Exact Test was used to test for differences in proportion of race due to small sizes within cells. Figure S1. Age and IQ distributions in the primary sample. Figure S2. Age and IQ distributions in the replication sample. Figure S3. Volume of cerebellar white matter in original and reprocessed images in the primary sample. Low reliability of this measure is clearly driven by one subject. Figure S4. Ratio of gray to white matter in the primary sample. Table S2 part 1. CHOP models with effects of IQ, Age, Sex, Diagnosis, IQ*Diagnosis, Age*Diagnosis, Sex*Diagnosis. Table S2 part 2. CHOP models with effects of IQ, Age, Sex, Diagnosis, IQ*Diagnosis, Age*Diagnosis, Sex*Diagnosis. Figure S5. Relationships of parental education with (a) brain volume and (b) IQ, in the subset of the CHOP sample for which parental education was available. Within the TDC sample, a positive relationship was observed between parent education and both TBV and IQ. Within the ASD group, the positive relationship between parent education and IQ was attenuated, and the relationship with TBV was reversed. Table S4. Yale models with effects of IQ, Age, Diagnosis, IQ*Diagnosis, Age*Diagnosis. [file 13229_2020_353_MOESM1_ESM.docx]

**Supplementary Material**

**Methods**

**Parental Education.** Within the primary sample, information on educational attainment was reported for each parent at six levels (e.g. “Some High School,” “High School,” etc., see Table 1). This categorical information was transformed into rank-order values (e.g. “Some High School” = 0; “High School” = 1, etc.). For each participant, mothers’ and fathers’ values were averaged to create a single parental education indicator. The distribution of these values was approximately normal (skewness: -0.48, Pearson’s kurtosis: 3.03). Therefore, although this derived variable is not truly an interval variable, we include it in ordinary least squares regression models. To address potential concerns about this treatment of this variable, we replicate findings from such models by treating each parent’s education as a binary factor (no college degree, or college degree or higher). The ASD and TDC groups did not differ on the mean level of parental education (*t* = -0.86, *p* = 0.39).

**Reliability of Image Processing Pipeline.** To assess reliability of the image-processing pipeline, 14 scans were randomly selected from the primary dataset, and re-processed (including skull stripping and Freesurfer edits). The dice coefficient of the resulting brain masks was 0.9994. The intra-class coefficient (ICC, two-way agreement) was calculated for each hemisphere for each measure reported here. ICC was good or better (above 0.75) for all measures except the cerebellum white matter, which was moderate. Low reliability on this measure was influenced by one outlier (see Figure S3). When this outlier was removed, reliability was excellent (ICC=0.99).

**Volume Definitions.** Freesurfer’s volume estimates were used as the volume measures.  Total brain volume (TBV) represents all voxels in the brain except ventricles, CSF, and choroid plexus.  Gray matter volume (GMV) is the sum of sub-cortical, cortical, and cerebellar gray matter.  Cortical white matter is the volume inside the white/gray surface, excluding non-white matter voxels.  We defined white matter volume (WMV) as the sum of cortical and cerebellar white matter.  We also examined cortical and cerebellar WMV and GMV separately.

|  | **ASD** | | **TDC** | | ***t*- value** | ***p-*value** |
| --- | --- | --- | --- | --- | --- | --- |
| Height inches (SD)  N  Range | 59.9 (6.06)  146  44.5-73.4 | | 59.7 (7.2)  135  43.4-76.2 | | -0.06 | 0.95 |
| Race | White: 206  Black: 10  Asian: 5  Biracial/other: 10  Not reported: 9 | | White: 145  Black: 46  Asian: 3  Biracial/other: 12  Not reported: 10 | |  | <0.001 |
| Parental Education  % (N)  Some High School:  High School:  Some College/2 Year Degree/Associate’s Degree:  4 Year College Degree/Bachelor’s Degree:  Master’s Degree/Graduate Degree:  Doctoral Degree:  Not Reported: | Mothers  5.8% (14)  0% (0)  18.3% (44)  31.7% (76)  16.3% (39)  2.5% (6)  25.4% (61) | Fathers  0.8% (2)  10.8% (26)  12.1% (29)  29.6% (71)  13.8% (33)  3.3% (8)  29.6% (71) | Mothers  8.3% (18)  0% (0)  11.1% (24)  25.0% (54)  23.1% (50)  3.7% (8)  28.7% (62) | Fathers  0% (0)  11.6% (25)  13.0% (28)  22.2% (48)  17.6% (38)  3.7% (8)  32.9% (69) |  |  |

Table S1. Height, race, and parental education for the primary sample. Fisher’s Exact Test was used to test for differences in proportion of race due to small sizes within cells.


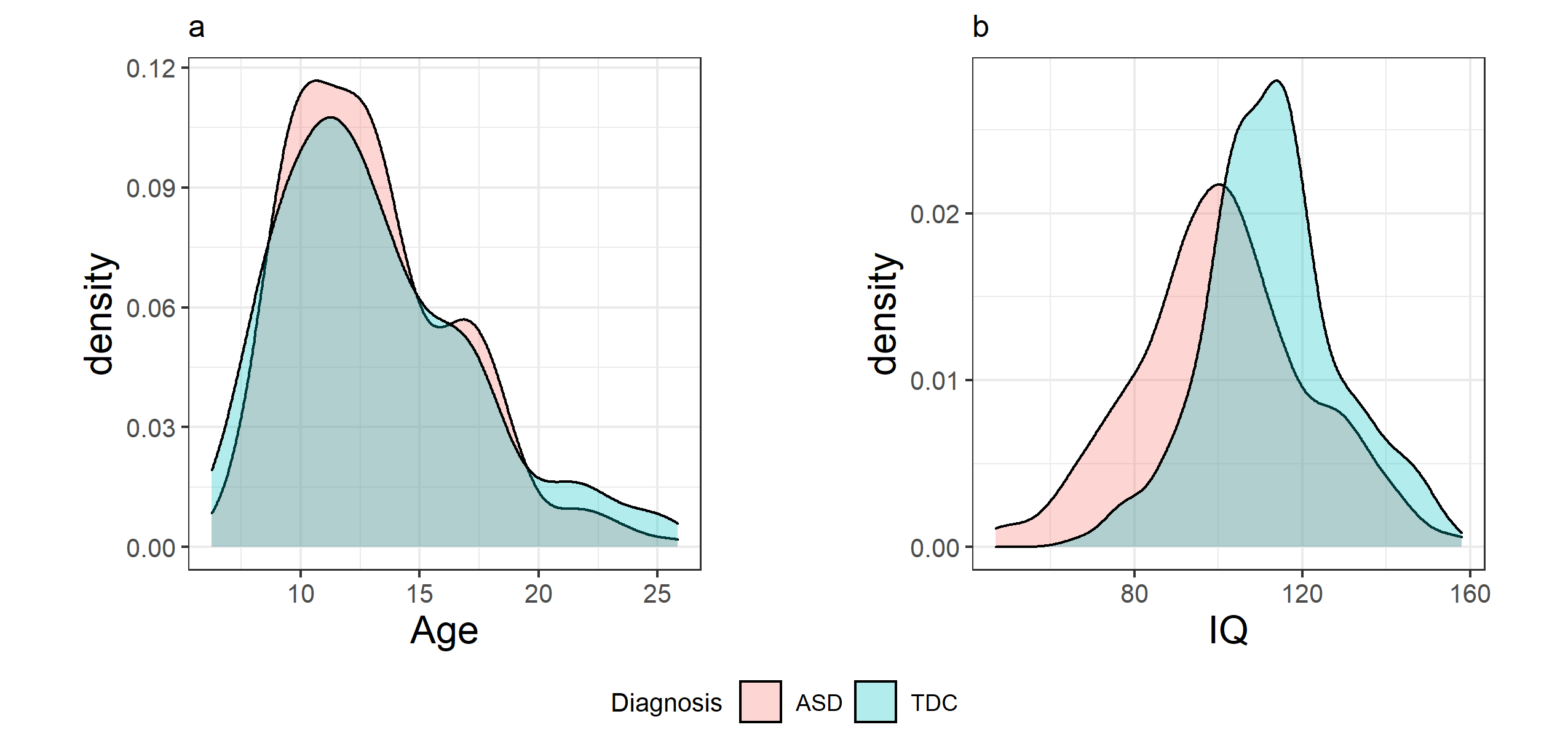
Figure S1. Age and IQ distributions in the primary sample.


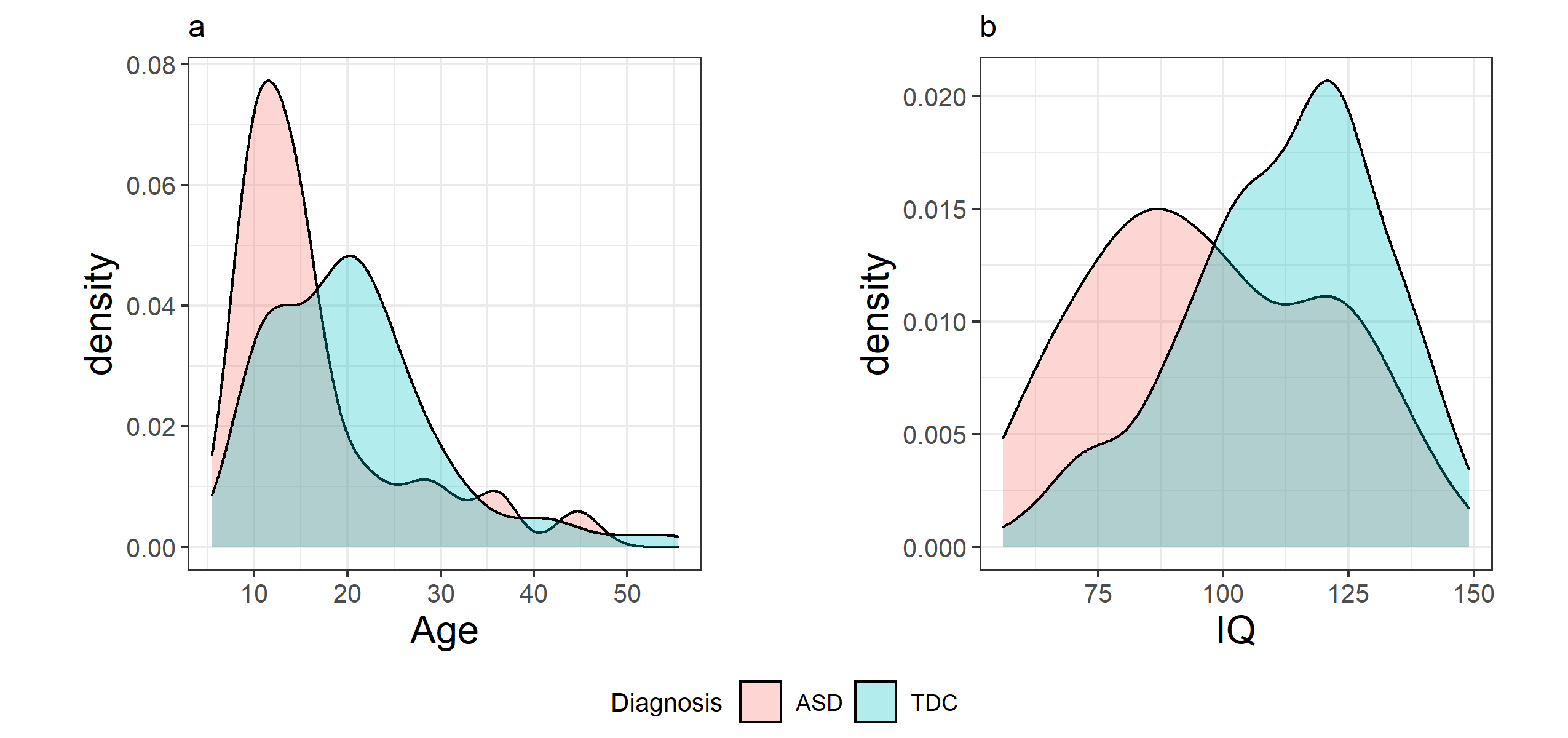


Figure S2. Age and IQ distributions in the replication sample.


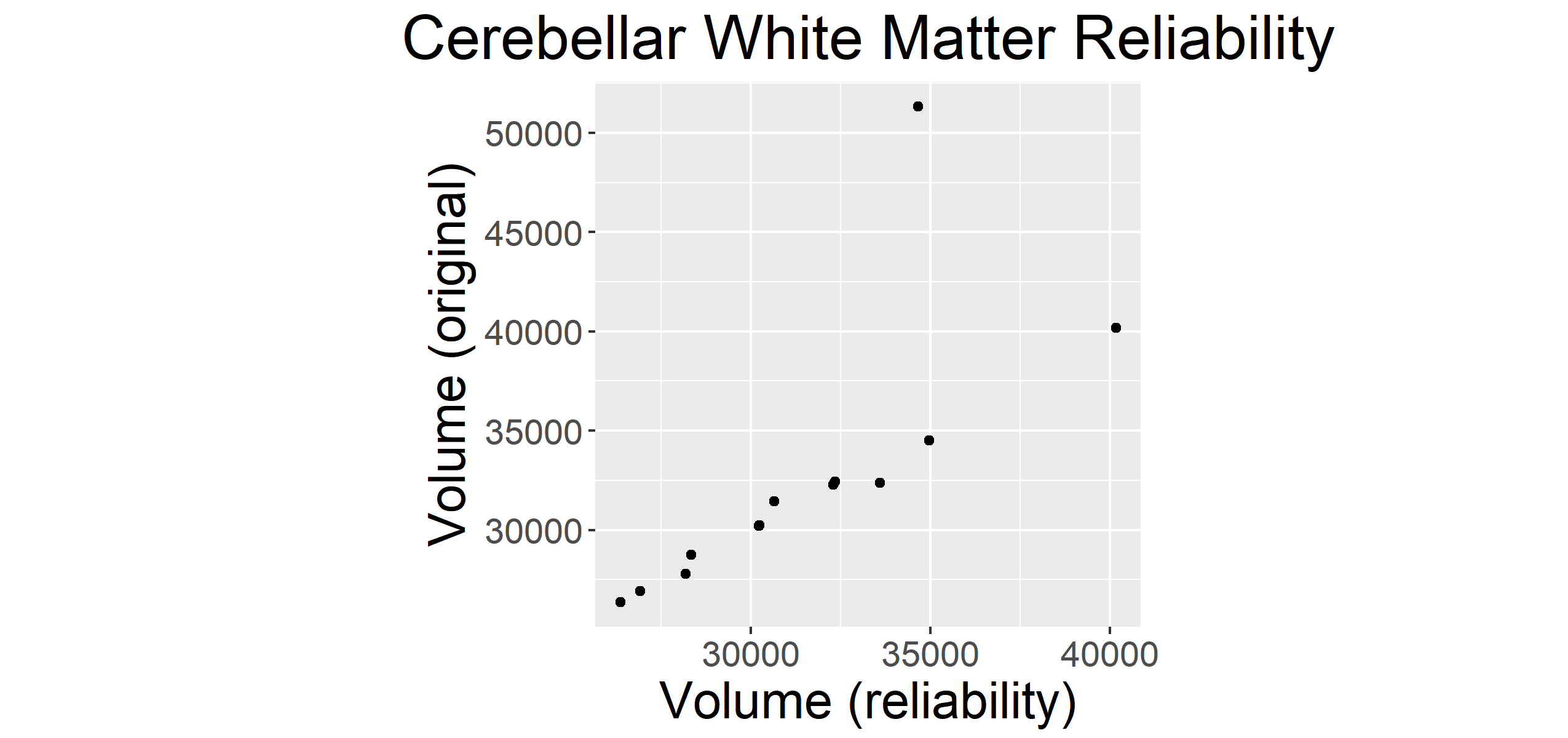


Figure S3. Volume of cerebellar white matter in original and reprocessed images in the primary sample. Low reliability of this measure is clearly driven by one subject.


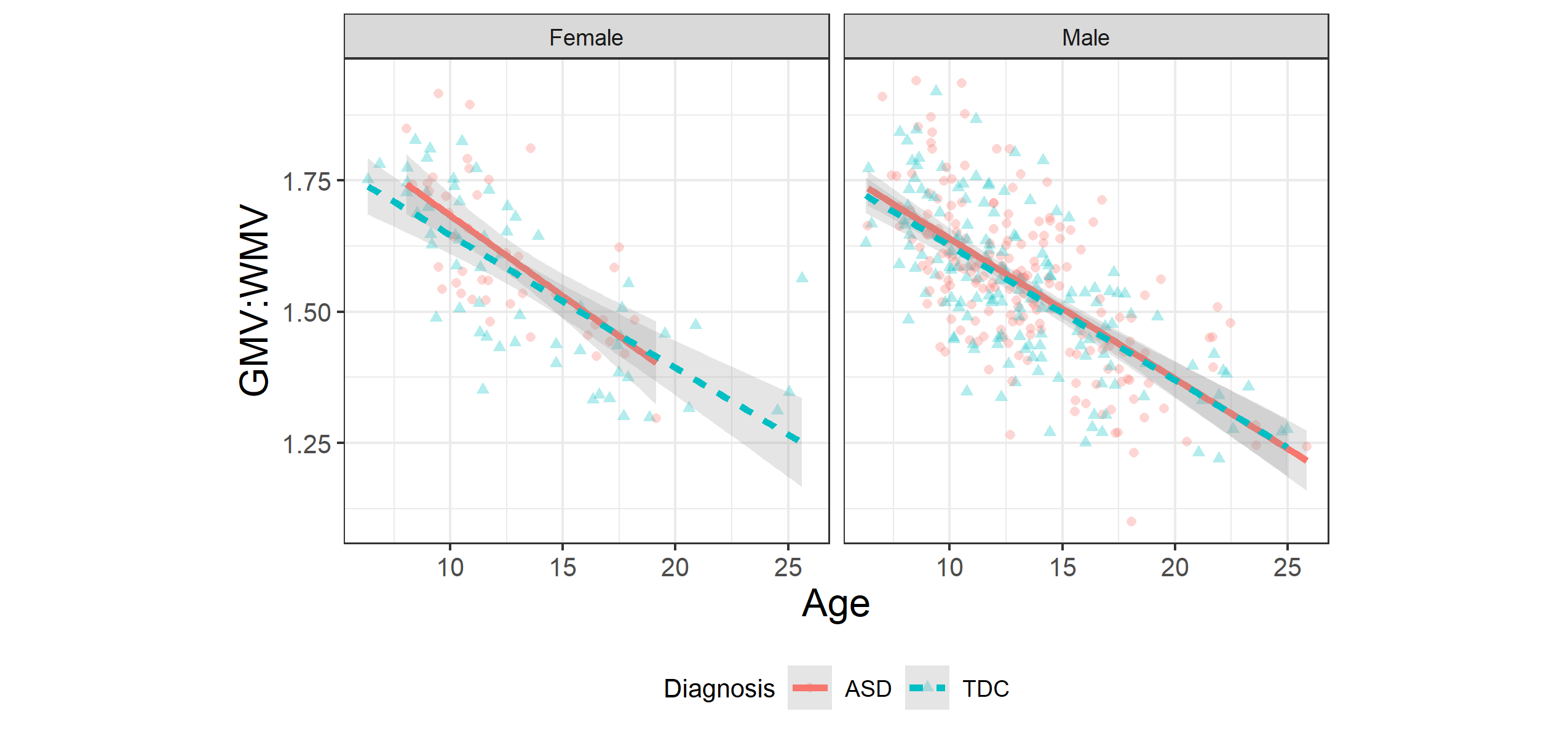
Figure S4. Ratio of gray to white matter in the primary sample.

CHOP models with effects of IQ, Age, Sex, Diagnosis, IQ*Diagnosis, Age*Diagnosis, Sex*Diagnosis (part 1)

|  | **TBV** | | | | **GMV** | | | | **WMV** | | | |
| --- | --- | --- | --- | --- | --- | --- | --- | --- | --- | --- | --- | --- |
| *Predictors* | *Estimates* | *std. Beta* | *p* | *partial η2* | *Estimates* | *std. Beta* | *p* | *partial η2* | *Estimates* | *std. Beta* | *p* | *partial η2* |
| Intercept | 1034.06 | -0.36 | **<0.001** | 0.69 | 698.66 | -0.35 | **<0.001** | 0.76 | 336.89 | -0.30 | **<0.001** | 0.47 |
| IQ | 1.53 | 0.25 | **<0.001** | 0.07 | 0.90 | 0.25 | **<0.001** | 0.08 | 0.63 | 0.20 | **<0.001** | 0.05 |
| Age | -1.87 | -0.06 | 0.132 | 0.01 | -6.19 | -0.34 | **<0.001** | 0.15 | 4.29 | 0.27 | **<0.001** | 0.09 |
| Sex | 86.00 | 0.73 | **<0.001** | 0.20 | 48.67 | 0.69 | **<0.001** | 0.21 | 37.32 | 0.62 | **<0.001** | 0.15 |
| Diagnosis | -210.61 | -0.22 | **<0.001** | 0.04 | -137.12 | -0.25 | **<0.001** | 0.06 | -73.36 | -0.15 | **0.002** | 0.02 |
| IQ*Diagnosis | 1.69 | 0.28 | **<0.001** | 0.04 | 1.06 | 0.29 | **<0.001** | 0.05 | 0.63 | 0.21 | **0.001** | 0.02 |
| Age*Diagnosis | 0.34 | 0.01 | 0.846 | 0.00 | 0.54 | 0.03 | 0.582 | 0.00 | -0.19 | -0.01 | 0.832 | 0.00 |
| Sex*Diagnosis | -7.75 | -0.07 | 0.498 | 0.00 | -4.14 | -0.06 | 0.516 | 0.00 | -3.64 | -0.06 | 0.533 | 0.00 |
| R^2^ / R^2^ adjusted | 0.291 / 0.280 | | | | 0.378 / 0.369 | | | | 0.281 / 0.269 | | | |
|  | **Cortical GMV** | | | | **Cortical WMV** | | | |  |  |  |  |
| *Predictors* | *Estimates* | *std. Beta* | *p* | *partial η2* | *Estimates* | *std. Beta* | *p* | *partial η2* |  |  |  |  |
| Intercept | 533.13 | -0.33 | **<0.001** | 0.72 | 313.46 | -0.30 | **<0.001** | 0.46 |  |  |  |  |
| IQ | 0.75 | 0.25 | **<0.001** | 0.07 | 0.61 | 0.21 | **<0.001** | 0.05 |  |  |  |  |
| Age | -5.70 | -0.37 | **<0.001** | 0.17 | 3.81 | 0.26 | **<0.001** | 0.08 |  |  |  |  |
| Sex | 38.53 | 0.65 | **<0.001** | 0.18 | 35.69 | 0.63 | **<0.001** | 0.16 |  |  |  |  |
| Diagnosis | -116.76 | -0.24 | **<0.001** | 0.06 | -68.71 | -0.15 | **0.002** | 0.02 |  |  |  |  |
| IQ*Diagnosis | 0.92 | 0.30 | **<0.001** | 0.06 | 0.60 | 0.21 | **0.001** | 0.02 |  |  |  |  |
| Age*Diagnosis | 0.36 | 0.02 | 0.668 | 0.00 | -0.26 | -0.02 | 0.756 | 0.00 |  |  |  |  |
| Sex*Diagnosis | -2.72 | -0.05 | 0.618 | 0.00 | -2.94 | -0.05 | 0.596 | 0.00 |  |  |  |  |
| R^2^ / R^2^ adjusted | 0.371 / 0.361 | | | | 0.277 / 0.265 | | | |  |  |  |  |

Table S2 part 1. CHOP models with effects of IQ, Age, Sex, Diagnosis, IQ*Diagnosis, Age*Diagnosis, Sex*Diagnosis

CHOP models with effects of IQ, Age, Sex, Diagnosis, IQ*Diagnosis, Age*Diagnosis, Sex*Diagnosis (part 2)

|  | **Cerebellum** | | | | **Cerebellar GMV** | | | | **Cerebellar WMV** | | | |
| --- | --- | --- | --- | --- | --- | --- | --- | --- | --- | --- | --- | --- |
| *Predictors* | *Estimates* | *std. Beta* | *p* | *partial η2* | *Estimates* | *std. Beta* | *p* | *partial η2* | *Estimates* | *std. Beta* | *p* | *partial η2* |
| Intercept | 128.54 | -0.28 | **<0.001** | 0.65 | 105.11 | -0.29 | **<0.001** | 0.68 | 23.43 | -0.16 | **<0.001** | 0.35 |
| IQ | 0.13 | 0.17 | **<0.001** | 0.03 | 0.11 | 0.19 | **<0.001** | 0.04 | 0.02 | 0.06 | 0.169 | 0.00 |
| Age | -0.01 | -0.00 | 0.946 | 0.00 | -0.49 | -0.16 | **<0.001** | 0.03 | 0.48 | 0.36 | **<0.001** | 0.14 |
| Sex | 8.70 | 0.59 | **<0.001** | 0.13 | 7.08 | 0.61 | **<0.001** | 0.14 | 1.63 | 0.32 | **<0.001** | 0.04 |
| Diagnosis | -17.98 | -0.17 | **0.004** | 0.02 | -13.32 | -0.20 | **0.006** | 0.02 | -4.66 | -0.03 | **0.029** | 0.01 |
| IQ*Diagnosis | 0.12 | 0.16 | **0.015** | 0.01 | 0.09 | 0.15 | **0.022** | 0.01 | 0.03 | 0.13 | 0.053 | 0.01 |
| Age*Diagnosis | 0.18 | 0.05 | 0.440 | 0.00 | 0.11 | 0.04 | 0.556 | 0.00 | 0.07 | 0.06 | 0.356 | 0.00 |
| Sex*Diagnosis | -2.47 | -0.17 | 0.107 | 0.01 | -1.77 | -0.15 | 0.136 | 0.00 | -0.70 | -0.14 | 0.182 | 0.00 |
| R^2^ / R^2^ adjusted | 0.182 / 0.169 | | | | 0.217 / 0.205 | | | | 0.193 / 0.181 | | | |
|  | Lateral Ventricles | | | | Third Ventricles | | | | GMV:WMV Ratio | | | |
| *Predictors* | *Estimates* | *std. Beta* | *p* | *partial η2* | *Estimates* | *std. Beta* | *p* | *partial η2* | *Estimates* | *std. Beta* | *p* | *partial η2* |
| Intercept | 6.76 | -0.06 | **<0.001** | 0.03 | 0.70 | -0.08 | **<0.001** | 0.12 | 1.92 | 0.04 | **<0.001** | 0.86 |
| IQ | -0.00 | -0.00 | 0.957 | 0.00 | 0.00 | 0.02 | 0.630 | 0.00 | -0.00 | -0.02 | 0.663 | 0.00 |
| Age | 0.27 | 0.18 | **<0.001** | 0.03 | 0.00 | 0.01 | 0.901 | 0.00 | -0.03 | -0.68 | **<0.001** | 0.46 |
| Sex | 0.46 | 0.08 | 0.326 | 0.00 | 0.03 | 0.12 | 0.126 | 0.01 | -0.02 | -0.13 | **0.031** | 0.01 |
| Diagnosis | -6.42 | -0.18 | **0.017** | 0.01 | -0.07 | -0.18 | 0.575 | 0.00 | -0.05 | -0.07 | 0.360 | 0.00 |
| IQ*Diagnosis | 0.03 | 0.10 | 0.152 | 0.00 | 0.00 | 0.11 | 0.115 | 0.01 | 0.00 | 0.03 | 0.631 | 0.00 |
| Age*Diagnosis | 0.16 | 0.10 | 0.118 | 0.01 | -0.01 | -0.16 | **0.016** | 0.01 | 0.00 | 0.03 | 0.540 | 0.00 |
| Sex*Diagnosis | 0.45 | 0.08 | 0.496 | 0.00 | -0.02 | -0.09 | 0.439 | 0.00 | 0.01 | 0.04 | 0.618 | 0.00 |
| R^2^ / R^2^ adjusted | 0.060 / 0.045 | | | | 0.046 / 0.031 | | | | 0.465 / 0.457 | | | |

Table S2 part 2. CHOP models with effects of IQ, Age, Sex, Diagnosis, IQ*Diagnosis, Age*Diagnosis, Sex*Diagnosis


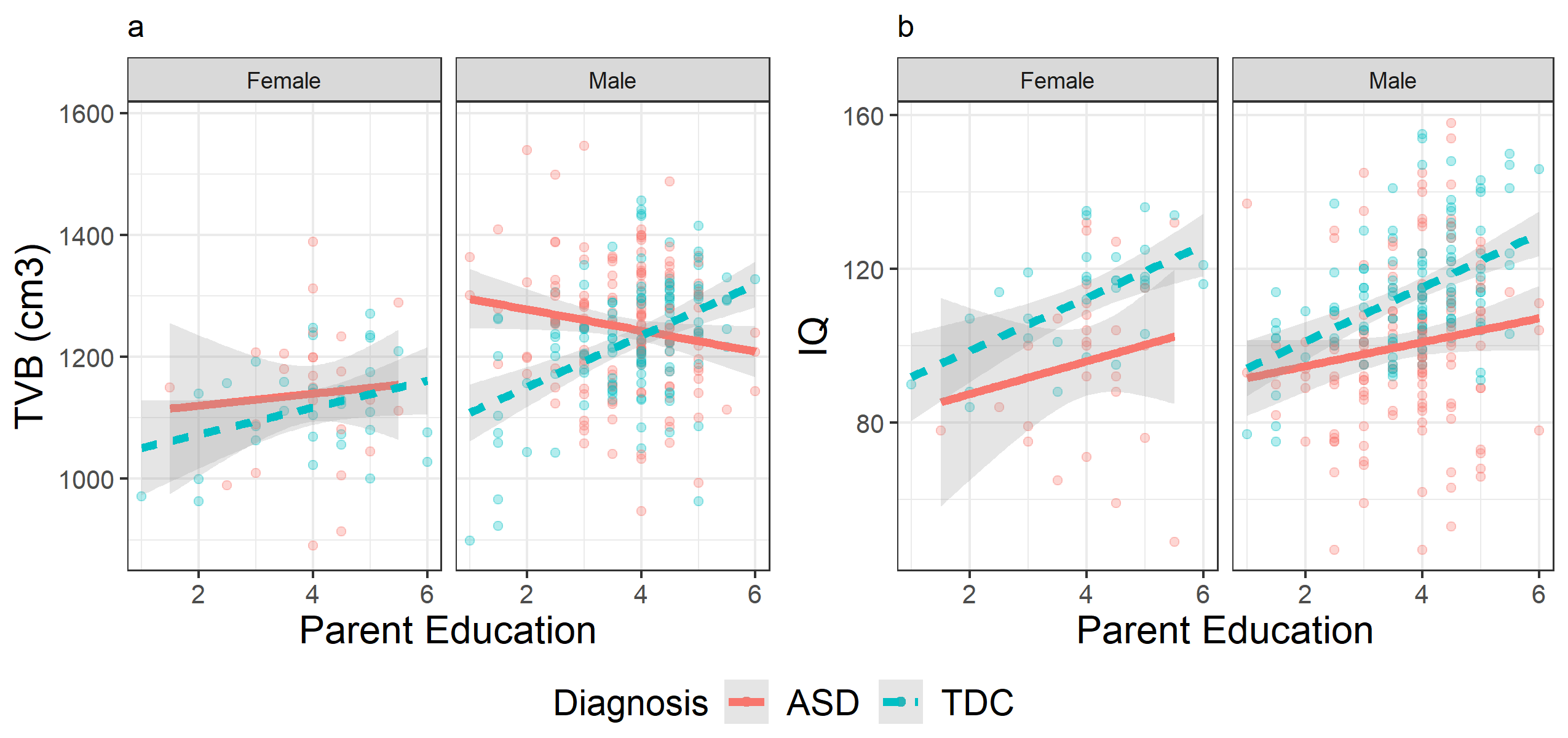


Figure S5. Relationships of parental education with (a) brain volume and (b) IQ, in the subset of the CHOP sample for which parental education was available. Within the TDC sample, a positive relationship was observed between parent education and both TBV and IQ. Within the ASD group, the positive relationship between parent education and IQ was attenuated, and the relationship with TBV was reversed.

|  | **Large** | **Small** | **Typical** | ***F*-value** | ***p*-value** |
| --- | --- | --- | --- | --- | --- |
| N | 10 | 10 | 220 |  |  |
| Males N (%) | 7 (70%) | 7 (70%) | 183 (83.2%) |  |  |
| Age (SD) | 15.04 (5.42) | 15.13 (4.69) | 12.79 (3.33) | 3.95 | 0.02 |
| Range | 8.33-25.86 | 9.16-21.7 | 6.36-23.6 |  |  |
| IQ (SD) | 98.7 (25.56) | 103.2 (15.84) | 100.85 (20.61) | 0.12 | 0.88 |
| Range | 49-140 | 73-127 | 47-158 |  |  |
| ADOS CSS | 7.8 (1.62) | 7.6 (1.9) | 6.86 (2.24) | 1.34 | 0.26 |
| Range | 6-10 | 3-10 | 1-10 |  |  |
|  |  |  |  |  |  |

Table S3. CHOP sample sex, age, IQ, and ADOS calibrated severity scores for ASD individuals categorized as having a brain greater than 2 SD above the mean for their age and sex (“large”), greater than 2 SD below the mean for their age and sex (“small”), or within 2 SD of the mean (“typical").

Yale IQ, Age, Diagnosis, IQ*Diagnosis, Age*Diagnosis

| *Predictors* | *Estimates* | *std. Beta* | *p* | *partial η2* | | *Estimates* | *std. Beta* | *p* | *partial η2* | *Estimates* | | *std. Beta* | | *p* | | *partial η2* |
| --- | --- | --- | --- | --- | --- | --- | --- | --- | --- | --- | --- | --- | --- | --- | --- | --- |
|  | **TBV** | | | | | **GMV** | | | | **WMV** | | | | | | |
| Intercept | 1175.21 | 0.19 | **<0.001** | | 0.78 | 757.18 | 0.18 | **<0.001** | 0.82 | 418.40 | 0.14 | | **<0.001** | | 0.62 | |
| IQ | 1.25 | 0.26 | **0.011** | | 0.04 | 0.54 | 0.17 | **0.046** | 0.02 | 0.71 | 0.28 | | **0.006** | | 0.04 | |
| Age | -3.82 | -0.32 | **0.002** | | 0.05 | -4.89 | -0.65 | **<0.001** | 0.23 | 1.06 | 0.17 | | 0.104 | | 0.02 | |
| Diagnosis | -139.69 | -0.43 | 0.101 | | 0.02 | -70.38 | -0.40 | 0.135 | 0.01 | -68.95 | -0.33 | | 0.121 | | 0.01 | |
| IQ*Diagnosis | 0.70 | 0.14 | 0.347 | | 0.01 | 0.17 | 0.05 | 0.686 | 0.00 | 0.53 | 0.21 | | 0.173 | | 0.01 | |
| Age*Diagnosis | 1.03 | 0.09 | 0.547 | | 0.00 | 1.31 | 0.17 | 0.168 | 0.01 | -0.28 | -0.05 | | 0.751 | | 0.00 | |
| R^2^ / R^2^ adjusted | 0.186 / 0.162 | | | | | 0.388 / 0.370 | | | | 0.160 / 0.135 | | | | | | |
|  | **Cortical GMV** | | | | | **Cortical WMV** | | | |  |  |  |  |  |  |  |
| Intercept | 588.92 | 0.19 | **<0.001** | | 0.80 | 394.78 | 0.14 | **<0.001** | 0.61 |  |  |  |  |  |  |  |
| IQ | 0.36 | 0.13 | 0.115 | | 0.01 | 0.67 | 0.28 | **0.007** | 0.04 |  |  |  |  |  |  |  |
| Age | -4.15 | -0.65 | **<0.001** | | 0.24 | 1.01 | 0.17 | 0.107 | 0.02 |  |  |  |  |  |  |  |
| Diagnosis | -61.06 | -0.42 | 0.121 | | 0.01 | -64.95 | -0.32 | 0.129 | 0.01 |  |  |  |  |  |  |  |
| IQ*Diagnosis | 0.17 | 0.06 | 0.624 | | 0.00 | 0.51 | 0.21 | 0.174 | 0.01 |  |  |  |  |  |  |  |
| Age*Diagnosis | 0.98 | 0.15 | 0.216 | | 0.01 | -0.30 | -0.05 | 0.724 | 0.00 |  |  |  |  |  |  |  |
| R^2^ / R^2^ adjusted | 0.404 / 0.387 | | | | | 0.155 / 0.130 | | | |  |  |  |  |  |  |  |
|  | **Cerebellum** | | | | | **Cerebellar GMV** | | | | **Cerebellar WMV** | | | | | | |
| Intercept | 131.88 | 0.09 | **<0.001** | | 0.71 | 108.26 | 0.05 | **<0.001** | 0.72 | 23.62 | 0.17 | | **<0.001** | | 0.53 | |
| IQ | 0.18 | 0.29 | **0.006** | | 0.04 | 0.13 | 0.26 | **0.011** | 0.04 | 0.04 | 0.27 | | **0.010** | | 0.04 | |
| Age | -0.48 | -0.32 | **0.004** | | 0.05 | -0.53 | -0.43 | **<0.001** | 0.09 | 0.05 | 0.12 | | 0.261 | | 0.01 | |
| Diagnosis | -7.87 | -0.22 | 0.482 | | 0.00 | -3.88 | -0.14 | 0.670 | 0.00 | -4.00 | -0.38 | | 0.185 | | 0.01 | |
| IQ*Diagnosis | -0.02 | -0.03 | 0.847 | | 0.00 | -0.04 | -0.08 | 0.617 | 0.00 | 0.02 | 0.12 | | 0.426 | | 0.00 | |
| Age*Diagnosis | 0.36 | 0.24 | 0.108 | | 0.02 | 0.34 | 0.28 | 0.063 | 0.02 | 0.02 | 0.05 | | 0.732 | | 0.00 | |
| R^2^ / R^2^ adjusted | 0.106 / 0.080 | | | | | 0.129 / 0.103 | | | | 0.121 / 0.095 | | | | | | |
|  | **Lateral Ventricles** | | | | | **Third Ventricles** | | | | **GMV:WMV Ratio** | | | | | | |
| Intercept | 6.27 | 0.30 | 0.071 | | 0.02 | 0.85 | 0.20 | **<0.001** | 0.22 | 1.77 | 0.02 | | **<0.001** | | 0.87 | |
| IQ | 0.06 | 0.19 | 0.063 | | 0.02 | -0.00 | -0.01 | 0.900 | 0.00 | -0.00 | -0.14 | | 0.056 | | 0.02 | |
| Age | 0.21 | 0.25 | **0.019** | | 0.03 | 0.01 | 0.22 | 0.050 | 0.02 | -0.01 | -0.77 | | **<0.001** | | 0.36 | |
| Diagnosis | 2.94 | -0.53 | 0.625 | | 0.00 | -0.13 | -0.41 | 0.546 | 0.00 | 0.03 | -0.04 | | 0.753 | | 0.00 | |
| IQ*Diagnosis | -0.09 | -0.26 | 0.095 | | 0.02 | 0.00 | 0.02 | 0.900 | 0.00 | -0.00 | -0.13 | | 0.242 | | 0.01 | |
| Age*Diagnosis | 0.12 | 0.14 | 0.342 | | 0.01 | -0.00 | -0.00 | 0.980 | 0.00 | 0.00 | 0.20 | | 0.072 | | 0.02 | |
| R^2^ / R^2^ adjusted | 0.165 / 0.140 | | | | | 0.070 / 0.042 | | | | 0.542 / 0.528 | | | | | | |

Table S4. Yale models with effects of IQ, Age, Diagnosis, IQ*Diagnosis, Age*Diagnosis

|  | **Large** | **Small** | **Typical** | ***F*-value** | ***p*-value** |
| --- | --- | --- | --- | --- | --- |
| N | 7 | 2 | 68 |  |  |
| Age (SD) | 13.17 (3.2) | 12.8 (0.71) | 15.47 (7.08) | 0.49 | 0.62 |
| Range | 10.1-19 | 12.3-13.3 | 8.2-44 |  |  |
| IQ (SD) | 103.14 (19.8) | 89.5 (30.41) | 96.79 (24.12) | 0.33 | 0.72 |
| Range | 75-132 | 68-111 | 56-144 |  |  |

Table S5. Yale sample age and IQ for ASD individuals categorized as having a brain greater than 2 SD above the mean for their age and sex (“large”), greater than 2 SD below the mean for their age and sex (“small”), or within 2 SD of the mean (“typical").


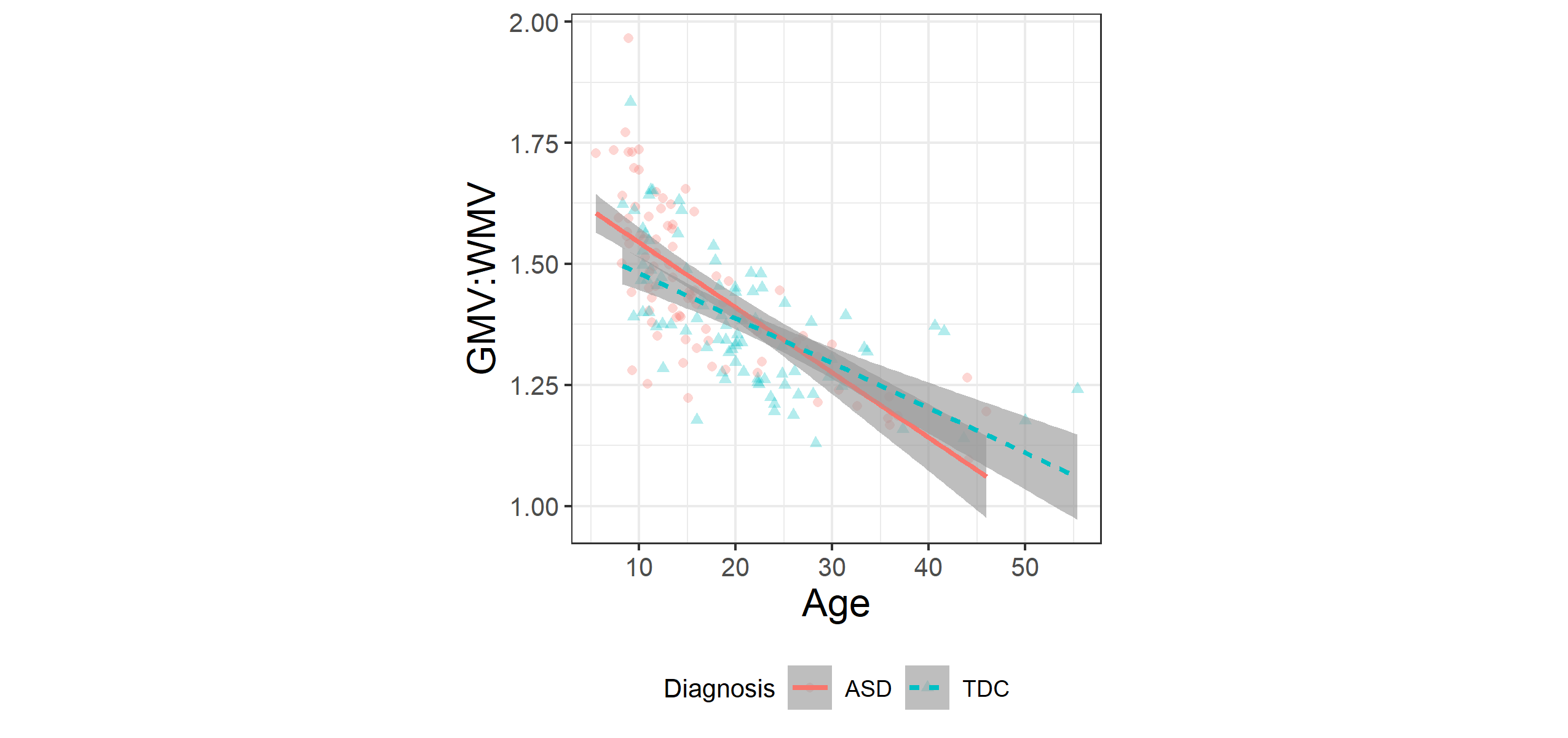
Figure S6. Ratio of gray to white matter in the replication sample.


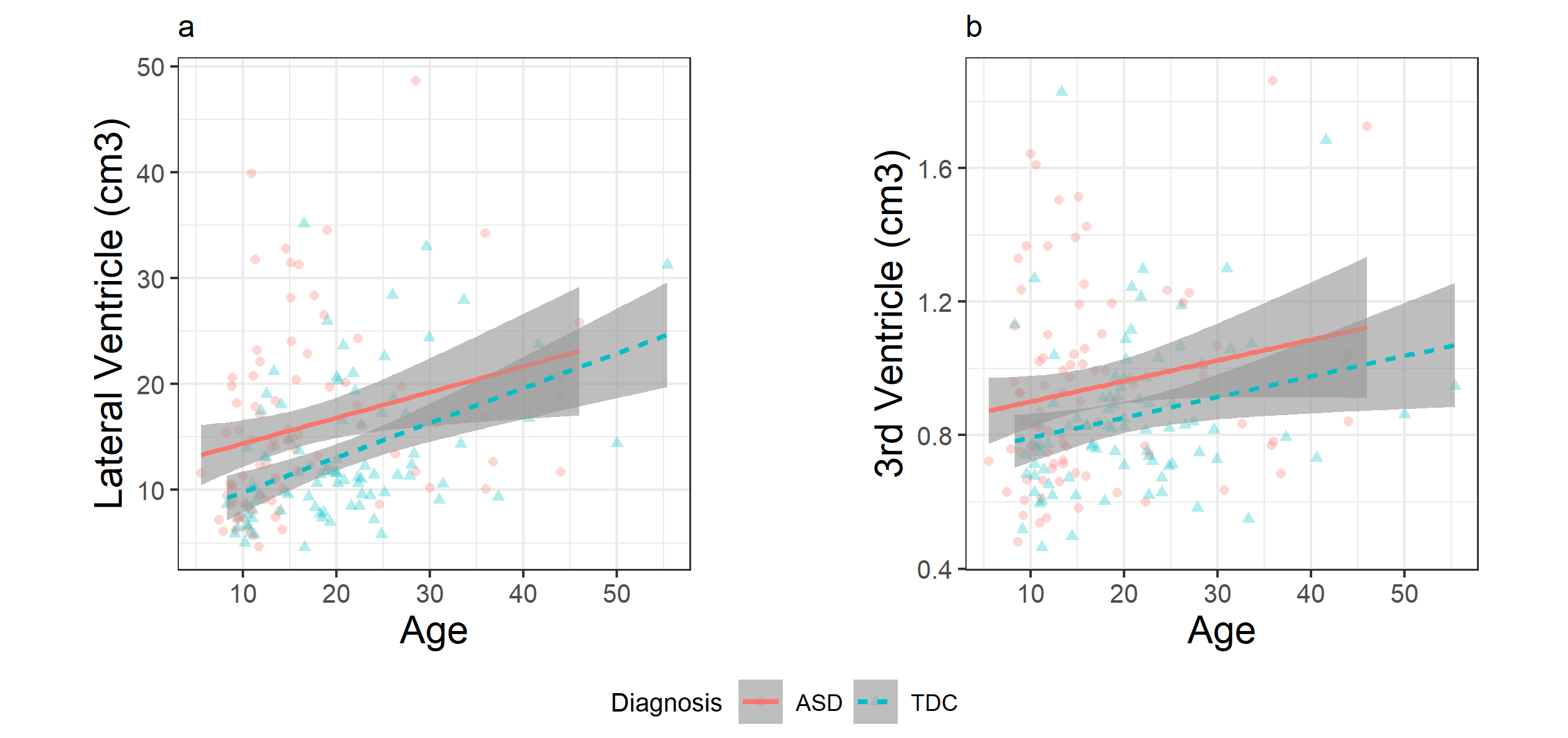


Figure S7. Ventricular volume in the replication sample.
